# The Prophages of *Citrobacter rodentium* Represent a Conserved Family of Horizontally Acquired Mobile Genetic Elements Associated with Enteric Evolution towards Pathogenicity

**DOI:** 10.1128/JB.00638-18

**Published:** 2019-04-09

**Authors:** Samuel J. Magaziner, Ziyue Zeng, Bihe Chen, George P. C. Salmond

**Affiliations:** a Department of Biochemistry, University of Cambridge, Cambridge, United Kingdom; Geisel School of Medicine at Dartmouth

**Keywords:** *Citrobacter rodentium*, attaching and effacing, bacteriophage genetics, bacteriophages, enteric pathogens, genetic exchange pathways, horizontal gene transfer, phylogenetic analysis, prophages, virulence determinants

## Abstract

Prophage-mediated horizontal gene transfer (HGT) plays a key role in the evolution of bacteria, enabling access to new environmental niches, including pathogenicity. Citrobacter rodentium is a host-adapted intestinal mouse pathogen and important model organism for attaching and effacing (A/E) pathogens, including the clinically significant enterohaemorrhagic and enteropathogenic Escherichia coli (EHEC and EPEC, respectively). Even though C. rodentium contains 10 prophage genomic regions, including an active temperate phage, ΦNP, little was known regarding the nature of C. rodentium prophages in the bacterium’s evolution toward pathogenicity. In this study, our characterization of ΦNP led to the discovery of a second, fully functional temperate phage, named ΦSM. We identify the bacterial host receptor for both phages as lipopolysaccharide (LPS). ΦNP and ΦSM are likely important mediators of HGT in C. rodentium. Bioinformatic analysis of the 10 prophage regions reveals cargo genes encoding known virulence factors, including several type III secretion system (T3SS) effectors. C. rodentium prophages are conserved across a wide range of pathogenic enteric bacteria, including EPEC and EHEC as well as pathogenic strains of Salmonella enterica, Shigella boydii, and Klebsiella pneumoniae. Phylogenetic analysis of core enteric backbone genes compared against prophage evolutionary models suggests that these prophages represent an important, conserved family of horizontally acquired enteric-bacterium-associated pathogenicity determinants. In addition to highlighting the transformative role of bacteriophage-mediated HGT in C. rodentium’s evolution toward pathogenicity, these data suggest that the examination of conserved families of prophages in other pathogenic bacteria and disease outbreaks might provide deeper evolutionary and pathological insights otherwise obscured by more classical analysis.

**IMPORTANCE** Bacteriophages are obligate intracellular parasites of bacteria. Some bacteriophages can confer novel bacterial phenotypes, including pathogenicity, through horizontal gene transfer (HGT). The pathogenic bacterium Citrobacter rodentium infects mice using mechanisms similar to those employed by human gastrointestinal pathogens, making it an important model organism. Here, we examined the 10 prophages of C. rodentium, investigating their roles in its evolution toward virulence. We characterized ΦNP and ΦSM, two endogenous active temperate bacteriophages likely important for HGT. We showed that the 10 prophages encode predicted virulence factors and are conserved within other intestinal pathogens. Phylogenetic analysis suggested that they represent a conserved family of horizontally acquired enteric-bacterium-associated pathogenic determinants. Consequently, similar analysis of prophage elements in other pathogens might further understanding of their evolution and pathology.

## Abstract (precis)

Bacteriophages are obligate intracellular parasites of bacteria. Some bacteriophages can confer novel bacterial phenotypes, including pathogenicity, through horizontal gene transfer (HGT). The pathogenic bacterium Citrobacter rodentium infects mice using mechanisms similar to those employed by human gastrointestinal pathogens, making it an important model organism. Here, we examined the 10 prophages of C. rodentium, investigating their roles in its evolution toward virulence. We characterized ΦNP and ΦSM, two endogenous active temperate bacteriophages likely important for HGT. We showed that the 10 prophages encode predicted virulence factors and are conserved within other intestinal pathogens. Phylogenetic analysis suggested that they represent a conserved family of horizontally acquired enteric-bacterium-associated pathogenic determinants. Consequently, similar analysis of prophage elements in other pathogens might further understanding of their evolution and pathology.

## INTRODUCTION

The nonmotile, Gram-negative, enteric bacterium Citrobacter rodentium is a natural host-adapted intestinal mouse pathogen, the causative agent of transmissible murine colonic hyperplasia, and an important model organism for the study of enteric pathogens of the attaching and effacing (A/E) family (1). C. rodentium was first isolated in 1972 from an outbreak of diarrhea in Swiss-Webster mice and then “rediscovered” by Schauer and Falkow in 1993 (2). It was originally classified as Citrobacter freundii biotype 4280 (ATCC 51459). There are two primary reference strains: DBS100 and ICC168 (2, 3). C. rodentium is a member of the A/E family of gastrointestinal pathogens. A/E members are characterized by the colonization of intestinal mucosa followed by the formation of so-called A/E lesions within the intestinal epithelium. These lesions are typified by the effacement of brush border microvilli and the formation of pedestal-like structures underneath the adherent bacterium. Lesion formation and epithelial distension are facilitated by a type III secretion system (T3SS) which injects effectors into infected epithelial cells, thereby reprogramming cell signaling, reorganizing cytoskeletal structures, and subverting native immune responses (4, 5).

The human enteric pathogens enteropathogenic Escherichia coli (EPEC) and enterohaemorrhagic E. coli (EHEC) are also members of the A/E family. EPEC is a major cause of infantile diarrhea, a leading source of high morbidity and mortality rates in developing countries (6). EHEC strains, notably serovar O157:H7, express the highly potent Shiga toxin (Stx), a causative agent of kidney failure, and are prevalent worldwide (7). Despite their clinical relevance, EPEC and EHEC have been difficult to study primarily due to their inability to infect mouse models. Importantly, it has been shown that EPEC and EHEC share a large portion of their genomes as well as their characterized proteomes with C. rodentium, including the locus of enterocyte effacement (LEE) pathogenicity island, which encodes several effector proteins and the T3SS required for A/E virulence (8, 9). Consequently, C. rodentium has become the standard for modeling human intestinal disease and A/E member pathogens such as EPEC and EHEC (10, 11).

The whole-genome sequencing of C. rodentium ICC168, the most widely studied reference strain, revealed that the bacterium’s genome is unstable as a result of repeat-mediated, large-scale genomic recombination due to the active transposition of mobile genetic elements such as prophages and that the bacterium probably represents a recently evolved pathogen of mice, possibly having emerged concurrently with the use of mouse models for human enteric diseases (12). In addition, the study of Petty et al. first identified a novel, active temperate bacteriophage, ΦNP, spontaneously released by C. rodentium (12).

With an estimated global 10^31^ phage particles, bacteriophages are thought to be the most abundant biological entities on Earth and are important drivers of bacterial evolution (13). The injected genomic DNA (gDNA) of some temperate phages can integrate into a specific locus of the host chromosome. This incorporated phage gDNA (termed a prophage) can then propagate through bacterial replication into populations of lysogen daughter cells until environmental cues stimulate excision and replication of the prophage element, directing the phages into the lytic cycle (14, 15). Prophage regions often act as mobile elements, and so the acquisition, alteration, and exchange of prophage-encoded traits through lysogeny represents a major source of horizontally mediated genomic flux within microbial populations. This mode of horizontal gene transfer (HGT) is often the primary step in the evolution of a bacterial species toward a pathogenic lifestyle, with several notable pathogens possessing key virulence traits encoded within prophage elements (16–18). In some instances, strains of EHEC have been shown to rely entirely on prophage induction to initiate host infection and renal disease (19). Genomic integration of foreign prophage elements can also impact the expression of native coding regions, altering phenotype and adaptively driving bacteria toward niches previously inaccessible or unfavorable (20, 21). Prophages can also serve as loci allowing incorporation and genetic exchange of so-called “fitness factors” (21). Fitness factors can enhance bacterial adaptation to environmental burdens, thereby allowing access to novel niches without necessarily contributing directly to pathogenesis. However, while prophages are known to be important for virulence phenotypes in other bacteria, they have not been studied in detail in C. rodentium.

C. rodentium genetics and mechanisms of LEE-facilitated host infection and interaction are well characterized (1). A recent study examined the spatiotemporal relationship of C. rodentium infection and the host microbiome, demonstrating a reliance on commensal gut microbiota for successful colonization of colonic mucosa (22). However, there is a paucity of information on the nature and importance of C. rodentium prophage-associated non-LEE virulence traits and genetic exchange in relation to host infection and the microbiome of the murine gut. Here, we present a detailed investigation of the prophages of C. rodentium ICC168, examining their biology and possible impact on the evolution of this A/E model organism toward pathogenicity. In addition to further examining the biology of ΦNP, we isolated and characterized a second, spontaneously released, functional temperate phage, ΦSM. We examined both ΦNP and ΦSM lysogen fitness and found evidence for nondeleterious prophage chromosomal integration and widely conserved prophage insertion site sequences across bacteria for both phages. We also identified the ΦNP and ΦSM bacterial surface receptors as lipopolysaccharide (LPS) residues, GlcII and GlcIII, respectively, common extracellular surface components in Gram-negative bacteria. We suggest that these temperate phages are likely key facilitators of HGT within C. rodentium and the enteric environment. We also provide evidence for functional gene loss and cargo gene acquisition, including several T3SS effectors, due to prophage integration possibly associated with C. rodentium’s virulence. Furthermore, we demonstrate the conservation of similar prophage regions in other known enteric pathogens and show that these conserved prophage loci are horizontally acquired, candidate virulence effector-containing elements and, conversely, not components of an ancestral enteric pathogen backbone. These observations provide new avenues for investigation of the non-LEE-associated pathogenesis determinants of C. rodentium and suggest that similar, systematic examination of prophage elements in other pathogenic bacteria, including strains associated with human disease outbreaks, might provide deeper evolutionary and pathological insights otherwise obscured by more classical backbone or 16S rRNA analysis.

## RESULTS

### Citrobacter rodentium spontaneously releases two temperate bacteriophages.

The genomes of C. rodentium ICC168 and DBS100 both contain 10 prophage regions, with 5 intact regions and 5 remnants of various degrees: ΦNP (44,907 bp), CRP28 (40,425 bp), CRP38 (36,759 bp), CRP49 (40,460 bp), CRP99 (37,185 bp), CRPr11 (7,975 bp), lambdoid remnant CRPr13 (10,555 bp), lambdoid remnant CRPr17 (6,476 bp), CRPr20 (19,000 bp), and CRPr33 (3,912 bp) (see Data Set S1 in the supplemental material). Despite evidence of induction and excision of CRP28, CRP38, CRP48, and CRP99 under standard LB growth conditions, only ΦNP had been previously reported to spontaneously generate viable temperate phage particles (12). That initial study characterized ΦNP as a member of the *Myoviridae*, identified its integration site as a 25-bp region corresponding to the 3′ terminal region of the tmRNA-encoding *ssrA* gene, and noted that the host range of ΦNP includes derivatives of Escherichia coli K-12, from which ΦNP lysogens could be isolated. This temperate phage may play a role in the natural genomic flux of C. rodentium and has potential for use in host strain engineering through synthetic biology methods. Consequently, we first reexamined the host range of ΦNP.

**FIG 1:**
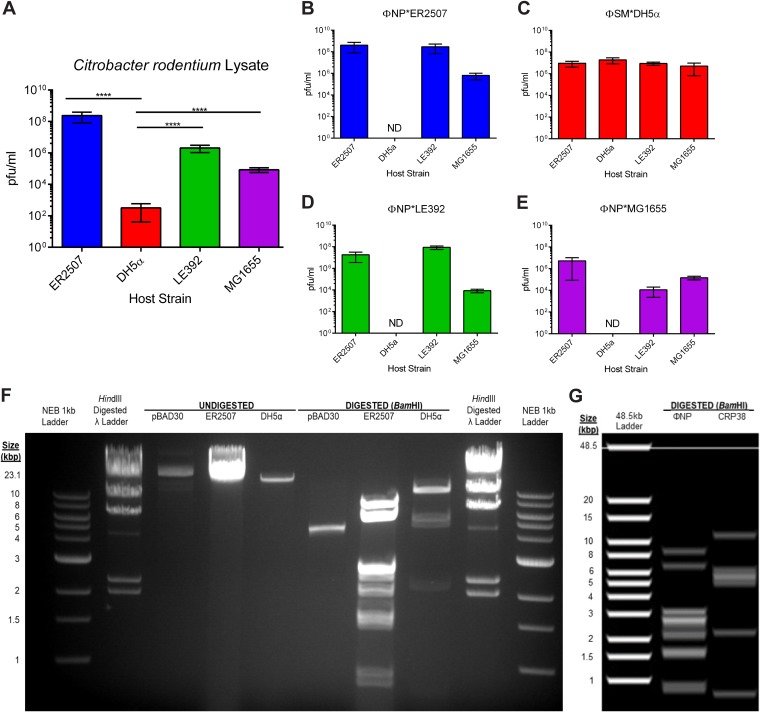
EOP assays of C. rodentium lysate reveal the presence of two temperate phages. (A) C. rodentium chloroform-treated supernatant from spun-down overnight cultures was titrated on soft-agar top lawns containing one of four E. coli K-12 strains, ER2507, DH5α, LE392, or MG1655, grown overnight at 37°C, and the number of PFU/milliliter was determined. Note the low apparent titer in tests conducted with DH5α. (****, *P* < 0.001, as determined by an unpaired *t* test). (B to E) Viral isolates were then single-plaque purified from the top lawns of each strain, increased to high titer, titrated back across the four K-12 variants, and grown overnight at 37°C, and the number of PFU/milliliter was determined. Phage collected from C. rodentium lysate-treated ER2507), LE392, and MG1655 strains were shown incapable of infecting DH5α (ND, not determinable). Conversely, phage isolated from DH5α top lawns was shown capable of infecting all four K-12 strains. (F) Genomic fingerprinting via restriction digestion with BamHI of phage gDNA extracted from ER2507 and DH5α phage isolates revealed two unique cut patterns. The ER2507 phage lysate cut pattern was consistent with that of ΦNP. The DH5α phage lysate cut pattern was consistent with that of CRP38 (later named ΦSM). (G) *In silico* genomic fingerprint following virtual BamHI digestion of prophages ΦNP and CRP38 (later named ΦSM). *In silico* genomic fingerprints of the other prophage regions can be found in Fig. S1 in the supplemental material. Displayed are means from triplicate EOP assays with error bars denoting standard deviations. In panels B to E, ΦNP/ΦSM*strain indicates the top lawn from which each viral stock was isolated. Gels were run using an NEB 1-kb ladder, HindIII-digested λ ladder, and digested/undigested pBAD30. The *in silico* gel was constructed using Geneious R11.

To investigate ΦNP host range, we titrated single plaque-purified ΦNP E. coli K-12 strain ER2507 lysates on a variety of *Gammaproteobacteria*, including six derivatives of E. coli K-12 representing commonly utilized laboratory strains (BW25113 and DH5α), well-studied wild-type (WT) strains (MG1655 and W3110), and strains shown to be highly permissive for phage ΦNP replication in earlier studies (ER2507 and LE392) (12) as well as on several strains of Serratia marcescens, *Serratia* sp. strain ATTC 39006, Salmonella enterica serovar Typhimurium 5383, Pantoea agglomerans 10Bp14, Citrobacter freundii Ballerup 7851, Pseudomonas aeruginosa PAO1, Kluyvera cryocrescens 2Kr27, Dickeya solani MK10, Yersinia enterocolitica, Pectobacterium atrosepticum SCRI-1043, and Photorhabdus luminescens subsp. *laumondii* TT01. Visible plaques formed only on K-12 derivatives ER2507, MG1655, W3110, and LE392. In contrast to the previous report (12), ΦNP did not form plaques on DH5α. To verify these observations and to exclude the possibility that restriction-modification was preventing infection, we conducted a two-round efficiency of plating (EOP) assay utilizing four derivatives of K-12 with chloroform-treated supernatant of an overnight C. rodentium culture instead of phages isolated from K-12 isolates (Fig. 1). The presence of restriction-modification would be marked by an increase of apparent titer when phages isolated from an initial host screen was used to reinfect the same host strain. In addition, decreases in apparent titers might be observed when other non-phage-derived K-12 host strains were infected.

Interestingly, we observed that plaques could form across all the E. coli strains tested, including DH5α, albeit at far lower efficiency (with an initial apparent titer of ∼10^3^ PFU/ml) (Fig. 1A). Despite intact restriction-modification, the E. coli K-12 strain MG1655 displayed a 1,000-fold greater susceptibility to plaque formation than DH5α. Phages from each K-12 strain were single-plaque purified and retested on each of the four strains (Fig. 1B to E). Phages that were isolated from ER2507, LE392, and MG1655 did not form plaques on DH5α (Fig. 1B, D, and E). Conversely, phages isolated from DH5α agar top lawns (Fig. 1C) were capable of forming plaques on all four K-12 strains. Moreover, the apparent reduction in titer first seen from titrating C. rodentium supernatant on DH5α was recovered (Fig. 1C), while EOPs of phages purified from ER207, LE392, and MG1655 were largely consistent with initial-round efficiencies (Fig. 1B, D, and E). Only phages isolated from MG1655 demonstrated a possible restriction-modification effect due to a decreased apparent viral titer of MG1655 harboring ΦNP [MG1655(ΦNP)] in ER2507 and LE392 hosts (Fig. 1C, ΦNP*MG1655). These results suggested that the inability of phages isolated from ER2507, LE392, or MG1655 to form plaques on DH5α was not a result of strain-specific mutations or restriction-modification but, more likely, indicated the presence of a second fully functional phage spontaneously released at a low titer into the supernatant by C. rodentium.

To test this hypothesis, we first analyzed high-titer phage lysates isolated from single plaques formed by titrating the C. rodentium supernatant on ER2507 and DH5α using phage genomic fingerprinting (Fig. 1F). Digestion with BamHI revealed two unique cut patterns for phage gDNA extracted from ER2507 lysates with respect to those extracted from DH5α. Virtual fingerprint gels were constructed for each of the intact and (because of its substantially intact nature) CRPr20 C. rodentium prophage regions to compare digestion patterns (Fig. 1G and Fig. S1). The fingerprint gDNA isolated from phage lysates propagated on ER2507 corresponded to the predicted digestion pattern of ΦNP. In contrast, the fingerprint of phage isolated from DH5α lysates corresponded to the predicted digestion pattern of prophage CRP38. To verify this identity, primers SM.P67 and SM.P69, corresponding to the 15,000- and 17,250-bp regions of CRP38, were used to amplify both the extracted phage gDNA as well as chromosomal DNA extracted from putative DH5α CRP38 lysogens. These PCR products were sequenced and confirmed to be amplified fragments of CRP38. This further showed that the second phage released by C. rodentium was the product of prophage region CRP38. This newly identified, fully functional temperate phage was named ΦSM.

**FIG 2:**
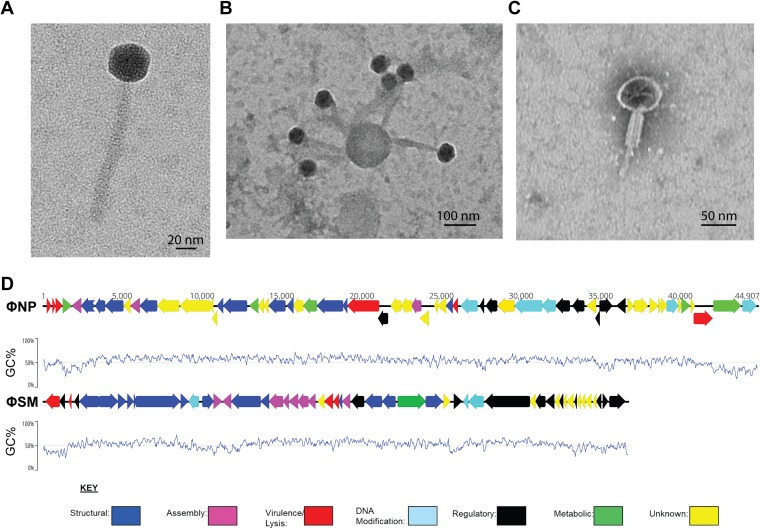
C. rodentium spontaneously releases two temperate phages, ΦNP and ΦSM. (A) TEM of the newly discovered ΦSM stained with uranyl acetate. Note the long noncontractile tail, characteristic of phage of the family *Siphoviridae*. (B) Grouping of ΦSM virions around a likely membrane element fragment; note the noncontracted tails even when virions are in the bound state. (C) TEM of the previously identified ΦNP stained with phosphotungstic acid. Note the short contracted tail characteristic of phage of the family *Myoviridae*. All ΦNP virions examined were seen in this contracted state. (D) Genetic organization of reannotated ΦNP and ΦSM showing GC content (window size, 100 bp). The clustering of coding domains with shared functionalities (structural, regulatory, etc.) allows more precise transcriptional control, and the swapping of such domains with other phage allows versatility and evolutionary advantage.

Using transmission electron microscopy (TEM), we examined the morphology of ΦSM (Fig. 2A). ΦSM virions had long, noncontractile tails even when in a bound state (Fig. 2B) and isometric heads, placing them within the order *Caudovirales* and family *Siphoviridae*, the same classification as the well-characterized phage λ (23). TEM of ΦNP lysates revealed the characteristic short, contractile tail associated with order *Caudovirales* and family *Myoviridae* members (Fig. 2C), such as the well-studied phage T4 (24). Following reannotation, the genetic organizations of both ΦNP and ΦSM were shown to exhibit functional modularity (Fig. 2D), common among bacteriophages (25).

While capable of forming plaques across the K-12 strains examined in this study, ΦSM was present in the supernatant of overnight cultures of C. rodentium at a significantly lower titer than ΦNP (∼10^3^ PFU/ml for ΦSM and ∼10^6^ to 10^8^ PFU/ml for ΦNP). Consequently, isolation of pure ΦSM from plaques generated on strains other than DH5α or its morphological characterization via TEM using the supernatant of C. rodentium proved difficult. Moreover, ΦSM was found to produce small, turbid, pinpoint plaques, indistinguishable from those produced by ΦNP (Fig. S2). These observations explained the initial oversight of the presence of ΦSM in earlier studies.

**FIG 3:**
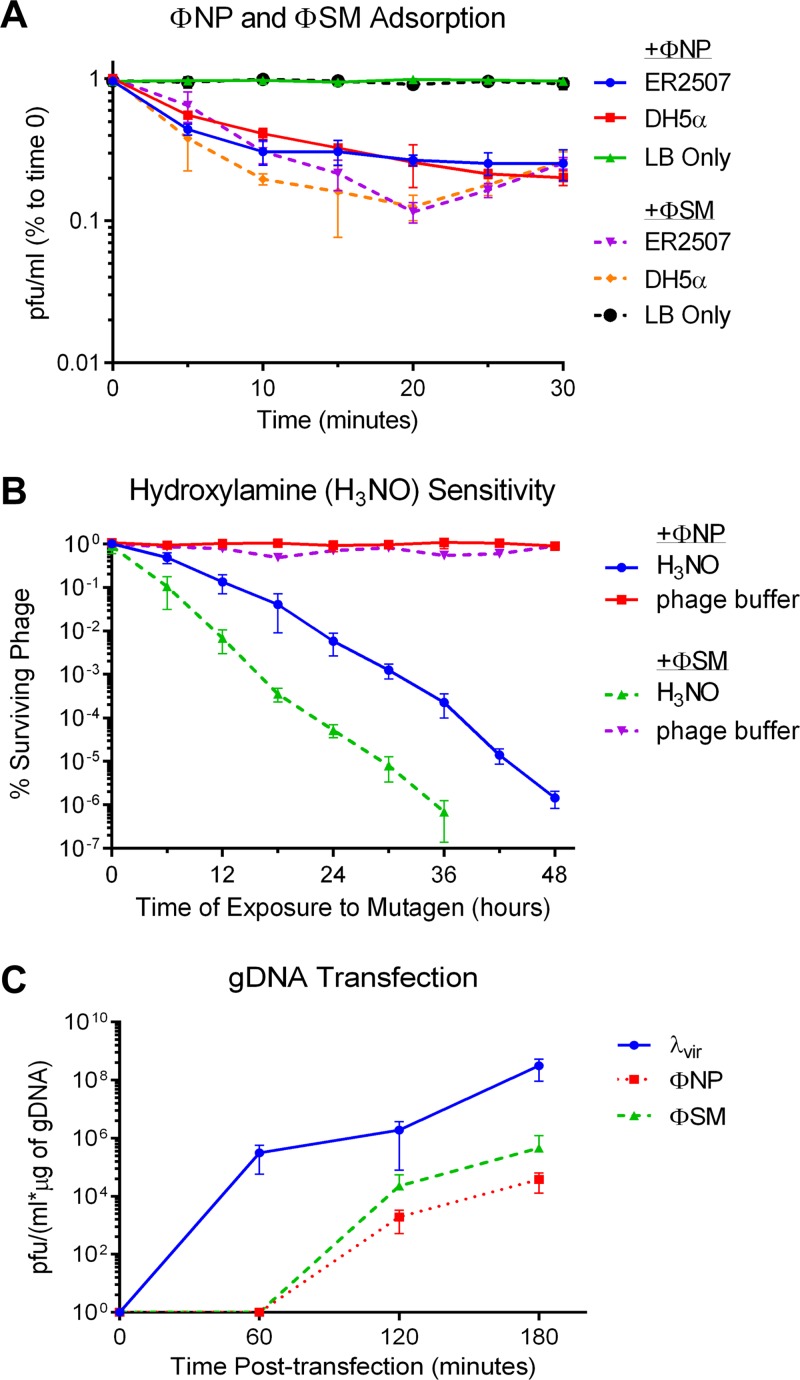
Characterization of ΦNP and ΦSM. (A) Representative graph showing the mean results from triplicate adsorption assays of ΦNP and ΦSM over 30 min onto E. coli K-12 strains DH5α and ER2507 with error bars denoting standard deviations. The *y* axis shows the amount of phage left unabsorbed as a percentage of phage at time zero. Despite ΦNP’s inability to form plaques onto DH5α, adsorption kinetics analogous to those of ΦSM suggest that plaque formation is prevented postinfection. (B) Killing curve constructed from titration of phage particles exposed to hydroxylamine (or phage buffer control) on ER2507 over time. The number of PFU/milliliter was normalized to that at time zero to determine the percent surviving phage for each sample. Note the higher rate of inactivation of ΦSM. (C) Graph showing the mean of triplicate experiments of transfection efficiency of gDNA isolated from ΦSM, ΦNP, and λ*vir*. No plaque**-**forming virions were isolated from ΦSM and ΦNP samples until 120 min.

Cultures made from colonies of putative lysogens, picked and purified from turbid spots on DH5α agar top lawns, spontaneously released phages into their supernatant and were immune to ΦSM superinfection in top agar spot tests, confirming ΦSM as a temperate phage. Strains lysogenic for ΦSM were isolated from E. coli K-12 strains BW25113, W3110, MG1655, LE392, DH5α, and ER2507. ΦNP and ΦSM proved to be heteroimmune, with ΦNP capable of infecting and lysogenizing ΦSM lysogens and *vice versa*. This allowed the construction of both single and dual ΦNP and ΦSM lysogens. DH5α strains lysogenic for ΦNP [DH5α(ΦNP)] could not be constructed by these standard methods. Interestingly, adsorption assays of free ΦNP and ΦSM using E. coli K-12 strains ER2507 and DH5α showed similar kinetics (Fig. 3A), suggesting that the inability of ΦNP to form plaques on DH5α may involve postinfection mechanisms such as issues with prophage induction or replication.

ΦNP and ΦSM were also differentiated by hydroxylamine sensitivity, with faster inactivation kinetics seen with ΦSM, consistent with its smaller (∼10 kbp) genome size (Fig. 3B). Both phages were capable of generating viable phage particles via gDNA transfection of chemically competent E. coli K-12 ER2507 cells (Fig. 3C). Last, the structural proteome of each phage was visualized by SDS-PAGE following polyethylene glycol (PEG) 8000 and CsCl gradient purification, with bands corresponding to the sizes of predicted structural proteins, some of which were verified by matrix-assisted laser desorption ionization (MALDI) fingerprinting (Fig. S3).

### ΦNP and ΦSM chromosomal integration is nondeleterious in E. coli K-12 hosts.

In addition to enabling horizontal genetic transfer, phages can also regulate bacterial populations through lytic action on prey species and fitness effects on lysogenized hosts (26, 27). Given C. rodentium’s adaptation to the murine intestine and the expected spontaneous release of two different temperate phages into the local gut microbiome, we were interested in assessing the effect of prophage integration on the fitness of lysogen and potential prey species, using E. coli as an appropriate host candidate.

**FIG 4:**
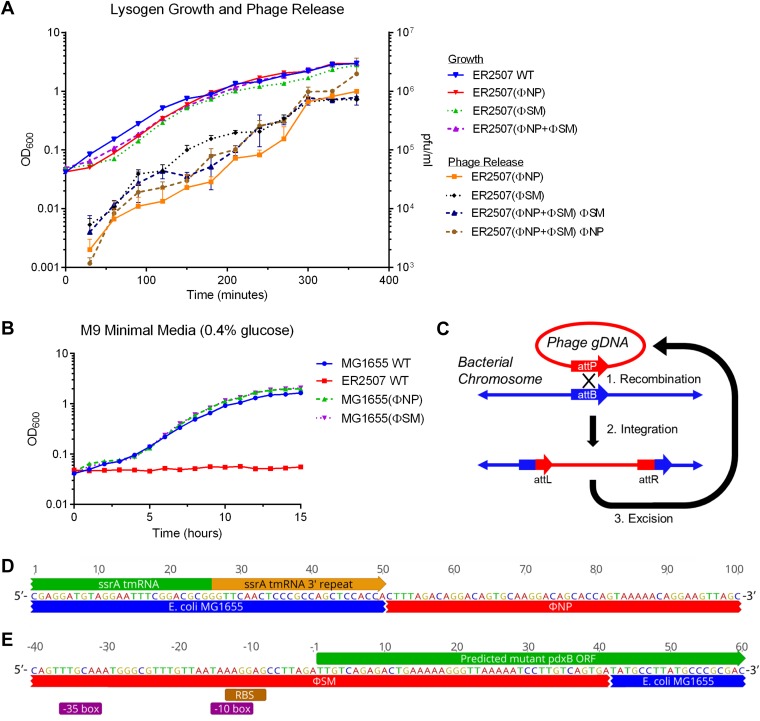
Integration of ΦNP and ΦSM is nondeleterious in K-12 hosts. (A) Growth of E. coli K-12 ER2507 WT and lysogen variants ER2507(ΦNP), ER2507(ΦSM), and ER2507(ΦNP+ΦSM) in LB at 37°C in a shaking water bath. Samples (200 μl) were taken at growth curve time points, chloroform treated, and titrated to determine phage release. In each case, the number of PFU/milliliter was found to increase proportionately with the OD_600_, with phage release showing no appreciable alteration to growth kinetics in the case of both single- and dual-lysogen species. For growth, the value shown is the mean from triplicate assays (standard deviations of <0.1). For phage release, the values shown are means from triplicate assays with error bars denoting standard deviations. Phage release for WT E. coli K-12 ER2507 was consistently undetectable (∼0 PFU/ml [data not shown]). Phage release of all other samples at time zero was also undetectable (∼0 PFU/ml [data not shown]). (B) Growth of E. coli K-12 MG1655 WT, ER2507 WT, MG1655(ΦNP), and MG1655(ΦSM) in M9 minimal liquid medium (with 0.4% glucose); values shown are the means from triplicate assays (standard deviations of <0.1). Phage integration proved nondeleterious to lysogen growth. (C) Schematic of prophage, recombination with, integration into, and excision from the bacterial host chromosome. (D) Genomic organization of ΦNP integration within MG1655 from the *attL* view. Prophage integration produces a duplication event of the 25-bp region corresponding to the 3′ terminal end of the *ssrA* tmRNA. (E) Genomic organization of ΦSM integration within MG1655 from the *attR* view. The 17-bp integration site corresponds to a region 6 bp within the 5′ terminal start of the *pdxB* gene. While insertion truncates this gene, a predicted, nearly constitutive Shine-Dalgarno sequence, Pribnow box (−10 box), and −35 box are directly upstream of a ΦSM-encoded TTG possible initiation codon, which reads into the native *pdxB* coding frame. Prophage DNA is denoted in red, host chromosomal DNA in blue, coding regions are shown in green, repeat regions are in orange, predicted RBS is shown in brown, and predicted regulatory units are shown in purple.

Growth of E. coli lysogens ER2507(ΦNP), ER2507(ΦSM), and ER2507 harboring both ΦNP and ΦSM [ER2507(ΦNP+ΦSM)] at 37°C in LB medium was recorded over 360 min with culture samples taken throughout growth for quantification of phage release (Fig. 4A). Growth of single and dual lysogens demonstrated an initial lag but demonstrated similar growth kinetics to the nonlysogenic ER2507 WT control. Phage release and accumulation were directly proportional to culture density, with phage titer leveling off in late exponential and early stationary phases. This suggested a phage-host equilibrium in which the spontaneous lysis of lysogenic cells reached a steady state with cell density and replication as seen in phage λ models (28). Next, growth of MG1655(ΦNP) and MG1655(ΦSM) was recorded at 37°C in M9 minimal medium (0.4% glucose) over 15 h (Fig. 4B). Growth of lysogens was similar to that of the MG1655 WT control. Taken together, these data suggested that ΦNP and ΦSM prophage integration and spontaneous induction had no discernible deleterious impacts in E. coli K-12 hosts under these growth conditions.

The phage attachment site (*attP*) of ΦNP DNA is a 25-bp (5′-TGGTGGAGCTGGCGGGAGTTGAACC-3′) sequence corresponding to the chromosomal insertion site (*attB*) comprising the 3′ terminal region of the *ssrA* gene in E. coli K-12 (Fig. 4C) (12). The *ssrA* gene encodes a transfer mRNA (tmRNA); this class of molecule can act as an intermediary between transfer RNAs (tRNA) and messenger RNAs (mRNA), serving to release stalled ribosomes from the end of defective mRNA with no stop codon (29). Integration of ΦNP DNA generates a repeat that reconstitutes an intact *ssrA* gene (Fig. 4D) (12). Using random-primed PCR (RP-PCR) and Sanger sequencing, we revalidated the integration site. Sequencing confirmed the same *ssrA* chromosomal insertion site across several lysogens, affirming prior results and demonstrating site conservation across multiple K-12 derivative strains.

Using similar methods, we found the *attP* site of ΦSM to be a 17-bp sequence (5′-ATCCTTGTTGATGAAAA-3′), corresponding to an *attB* 6 bp within the 5′ terminal start of the *pdxB* gene in E. coli K-12 (Fig. 4E). The gene *pdxB* encodes erythronate-4-phosphate dehydrogenase, the second catalyzing enzyme in the subpathway of pyridoxal 5′-phosphate (PLP) biosynthesis, which is itself part of cofactor vitamin B_6_ biosynthesis (30). E. coli contains at least 60 enzymes which utilize PLP, and E. coli mutants lacking PdxB are auxotrophic: they are unable to grow on M9 minimal medium with glucose as a sole carbon source (31). The insertion of the ΦSM phage DNA in E. coli K-12 truncates the *pdxB* coding region, generating a loss of the ATG (methionine) start codon and the AAA (lysine) codon and separation of the remaining reading frame from native regulatory elements upstream. No *pdxB* homologue is found within the ΦSM genome. However, further bioinformatic interrogation revealed a possible in-frame TTG (methionine/leucine) rare start codon located within the inserted prophage element 41 bp upstream of the truncated *pdxB* gene. From this TTG site, a near-consensus Shine-Dalgarno sequence (32) (5′-AAGGAG-3′) was found at the bp −7 position. In addition, utilizing a database of known phage-regulatory genomic regions, phiSITE (33), a near-consensus Pribnow box (or −10 box) (5′-TAAAGG-3′) was found at the bp −10 position with a second near-consensus promoter sequence (5′-TTGCAA-3′) located at the bp −31 position (Fig. 4E) (34). Despite the apparent truncation generated by the ΦSM prophage insertion, MG1655(ΦSM) strains were able to grow on minimal medium (Fig. 4B). This suggested that the ΦSM prophage insertion into the 5′ end of *pdxB* caused no functional loss of the cognate product.

Further analysis revealed that the 25-bp *ssrA* and 17-bp *pdxB* (or *traF* in C. rodentium; see below) integration sites and corresponding coding regions are widely conserved across bacteria (showing 8,349 unique matches for the ΦNP integration site and 3,668 unique matches for the ΦSM integration site with 100% base pair identity). Most sequence conservation for both ΦNP and ΦSM was found in species of the Enterobacteriaceae (including species of E. coli, *Salmonella, Shigella, Klebsiella,* and *Citrobacter*); unique taxon matches were also identified for each phage, such as bacteria of *Vibrionaceae* and *Pasteurellaceae* for ΦNP and of *Terrabacteria* and *Bacteroidetes* for ΦSM. In conjunction with nondeleterious phage infection and prophage integration maintaining host fitness, widespread conservation of integration sites (allowing access to a broader range of potential hosts) suggests that these phages evolved in such a way as to better facilitate HGT within the enteric environment.

### The receptor of ΦNP and ΦSM is LPS.

Bacteriophage host recognition and binding (adsorption) to a receptor are the first stage of phage infection and the primary limiting factor of phage propagation and host range. Receptors exploited by phages can be any component of the bacterial surface ranging from residues of the lipopolysaccharide (LPS) in Gram-negative bacteria (one of the most common receptors) to flagella, pili, or surface membrane proteins (35, 36). Some phages use a dual-receptor model of adsorption, such as phage T4 which requires both specific LPS residues as well as the outer membrane protein, OmpC, to infect (37, 38).

To provide insight into potential microbiome interactions and host range specificities, we examined the C. rodentium ΦNP and ΦSM host receptors. We constructed a library of randomly inserted transposon mutants of E. coli K-12 and screened them against ΦNP and ΦSM. Mutants displaying elevated resistance to infection were screened further, and the corresponding transposon insertion sites were identified. For both phages, sequencing revealed numerous mutants with transposon insertions in genes responsible for the enzymatic steps of core lipopolysaccharide (LPS) biosynthesis. E. coli LPS consists of three regions: (i) lipid A, the hydrophobic membrane anchor, (ii) a short core oligosaccharide (core OS, comprised of an inner and outer region), and (iii) a polymer of glycosyl units known as O polysaccharide (O-PS). E. coli K-12 lacks an O-PS (also called an O antigen), and the core OS is the outermost layer (39, 40). Each gene of the biosynthetic cluster is proposed to catalyze a specific glycosylation step of LPS core biosynthesis (Fig. S4).

To determine receptor specificity, we employed single in-frame LPS gene knockout mutants to assess EOP against both phages (Table 1) (38, 41). For both ΦNP and ΦSM, we noted highly resistant mutants. For both phages, the galactose (Gal) side-chain-deficient JW3603-2 (Δ*waaB*::*kan*) mutants showed no discernible resistance. For ΦNP, all other knockouts, except JW3601-3 (Δ*waaR*::*kan*), demonstrated total resistance (with EOPs of <10^−10^ PFU/ml). This suggested that glucose II (GlcII), the addition of which is catalyzed by the product of *waaO*, is an essential LPS residue for ΦNP infection. Interestingly, JW3601-3 (Δ*waaR*::*kan*) mutants demonstrated total resistance to ΦSM, as did all other LPS mutants (except the TY0703 Δ*waaO-waaB*::*cm* strains). This suggested that ΦSM, like ΦNP, relies on LPS for infection but utilizes a different set of residues, namely, GlcIII or GlcI (with no Gal side chain) for infection.

**TABLE 1:** EOP of ΦNP and ΦSM on LPS mutant strains^*a*^

Strain	ΦNP (PFU/ml)	ΦSM (PFU/ml)
BW25113 WT	1.0 ± 0.1	1.0 ± 0.14
JW3601-3 (*ΔwaaR*::*kan*)	1.1 ± 0.3	<10^−10^
JW3602-1 (*ΔwaaO*::*kan*)	<10^−10^	<10^−10^
JW3603-2 (*ΔwaaB*::*kan*)	0.95 ± 0.23	0.88 ± 0.12
TY0703 (*ΔwaaO-waaB*::*cm*)	<10^−10^	1.38 ± 0.28
JW3606-1 (*ΔwaaG*::*kan*)	<10^−10^	<10^−10^
TY0707 (*ΔwaaF*::*cm*)	<10^−10^	<10^−10^
TY0708 (*ΔwaaC*::*cm*)	<10^−10^	<10^−10^

a Shown are the means from triplicate EOP assays with standard deviations.

**FIG 5:**
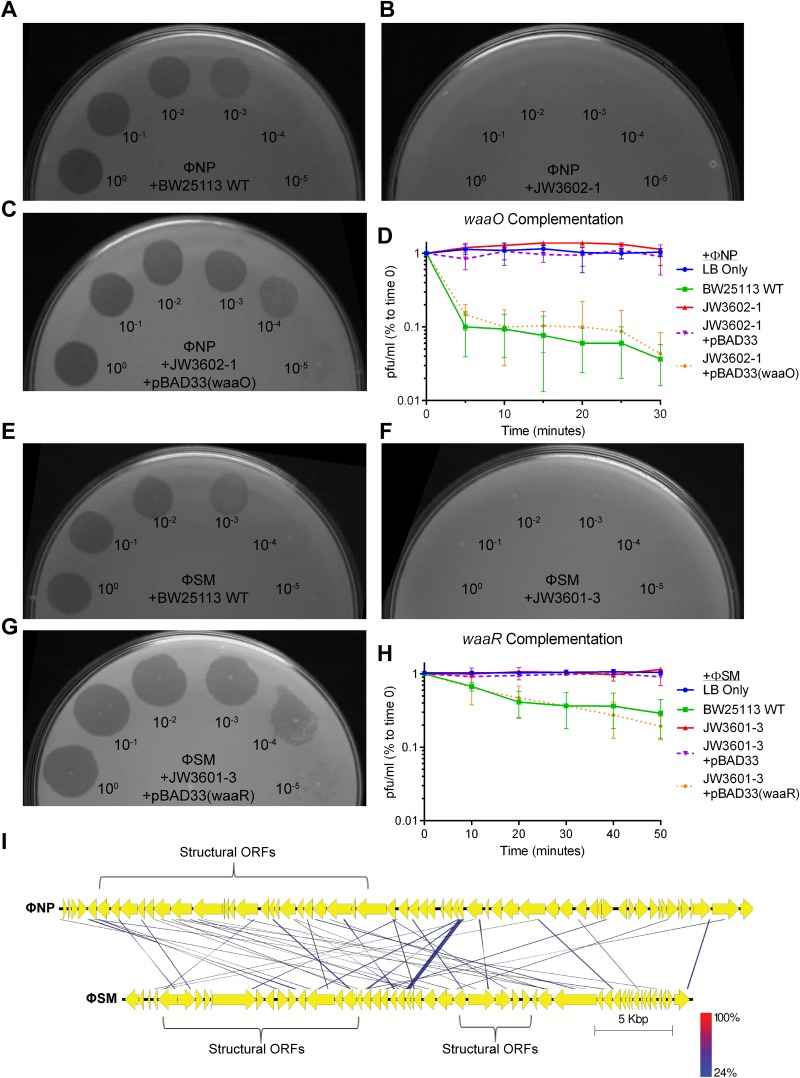
The extracellular receptors of ΦNP and ΦSM are LPS residues. (A to C) ΦNP spot tests with serially diluted phage (initial titer of ∼10^9^ PFU/ml) on strains BW25113 WT (A), JW3602-1 (Δ*waaO*::*kan*) (B), and complemented *waaO* in pBAD33 (C) on 0.1% arabinose-containing soft agar LB top lawn plates. Rescue of *waaO* restored phage sensitivity and plaque formation. (D) Representative graph showing the mean results of triplicate adsorption assays of ΦNP over 30 min on E. coli K-12 strains BW25113 WT, JW3602-1 (Δ*waaO*::*kan*), JW3602-1 (Δ*waaO*::*kan*) containing an empty arabinose-inducible plasmid, pBAD33, and a complemented JW3602-1 (Δ*waaO*::*kan*) strain containing pBAD33(*waaO*), with error bars denoting standard deviations. The left axis shows the amount of phage left unabsorbed as a percentage of the amount of phage at time zero. All samples were under 0.1% arabinose induction. Complementation of *waaO* restored phage binding. (E to G) ΦSM spot tests with serially diluted phage (initial titer of ∼10^9^ PFU/ml) on strains BW25113 WT (E), JW3601-3 (Δ*waaR*::*kan*) (F), and complemented *waaR* in pBAD33 (G) on 0.1% arabinose-containing soft agar LB top lawn plates. Rescue of *waaR* restored phage sensitivity and plaque formation. (H) Representative graph showing the mean results of triplicate adsorption assays of ΦSM over 50 min on E. coli K-12 strains BW25113 WT, JW3601-3 (Δ*waaR*::*kan*), JW3601-3 (Δ*waaR*::*kan*) containing an empty arabinose inducible plasmid pBAD33, and a complemented JW3601-3 (Δ*waaR*::*kan*) containing pBAD33(*waaR*), with error bars denoting standard deviations. The *x* axis shows the amount of phage left unabsorbed as a percentage of the amount of phage at time zero. All samples were under 0.1% arabinose induction. Complementation of *waaR* restored phage binding. (I) Whole alignment of reannotated ΦNP and ΦSM translated open reading frames conducted by tblastx. Little protein level homology is seen between the translated open reading frames (ORFs) of each phage. ORFs predicted to encode structural proteins are annotated. Lack of consensus ORFs suggests that ΦNP and ΦSM evolved convergently to utilize LPS as bacterial receptors. DNA is represented by black bars with coding regions as arrows. Similarity is indicated by a color scale for high (red) and low (blue) rates of translated amino acid matches.

Next, we conducted soft-agar top lawn spot and adsorption assays with complemented *waaO* in JW3602-1 for ΦNP and *waaR* in JW3601-3 for ΦSM (Fig. 5). Complementation of *waaO* in JW3602-1 restored ΦNP phage sensitivity, as determined by plaque formation (Fig. 5A to C). Similarly, rescue of *waaR* in JW3601-3 restored ΦSM phage sensitivity (Fig. 5E to G). LPS mutant strains exhibited little to no phage adsorption in both cases, with sugar transferase complementation restoring phage binding and kinetics to levels similar to those of the BW25113 WT (Fig. 5D and H). These data confirmed that ΦNP and ΦSM utilize residue-specific LPS components for adsorption and infection, with ΦNP utilizing the GlcII residue and ΦSM utilizing the GlcIII residue. Interestingly, the alignments of the translated open reading frames (ORFs) performed by tblastx of the genomes of ΦNP and ΦSM revealed little homology, suggesting convergent evolution toward LPS binding specificity (Fig. 5I).

In addition, tests were conducted on in-frame knockout mutants defective for other common phage receptors (Δ*fhuA*::*kan*, Δ*tsx*::*kan*, Δ*ompF*::*kan*, Δ*ompA*::*kan*, Δ*ompW*::*kan*, Δ*uidC*::*kan*, Δ*fadL*::*kan*, Δ*yicC*::*kan*, Δ*lamB*::*kan*, and Δ*ompC*::*kan* strains) with no drop in EOP observed. ΦNP and ΦSM were also tested against dual Δ*ompC*::*kan*-LPS::*cm* knockout mutants, showing no deviation of results gathered from single-LPS knockouts (EOPs consistent with those shown in Table 1).

### ΦSM and reservoir CRPr20: a possible means of overcoming functional gene loss.

Previous studies identified functional accessory and core gene loss due to 5 of 10 prophage integrations within the C. rodentium chromosome possibly responsible for evolution toward its pathogenic niche (12, 42). CRPr20 and CRP28 were identified as inserted within the Flag-1 and -2 flagellar biosynthetic gene clusters, respectively, suggesting a direct relationship between prophage integration and C. rodentium’s loss of motility. CRP49 was noted to have inserted into *gatD* (encoding galactitol-1-phosphate dehydrogenase), which is essential for the metabolism of galactitol, and C. rodentium was unable to grow with this as a sole carbon source (12, 43).

**FIG 6:**
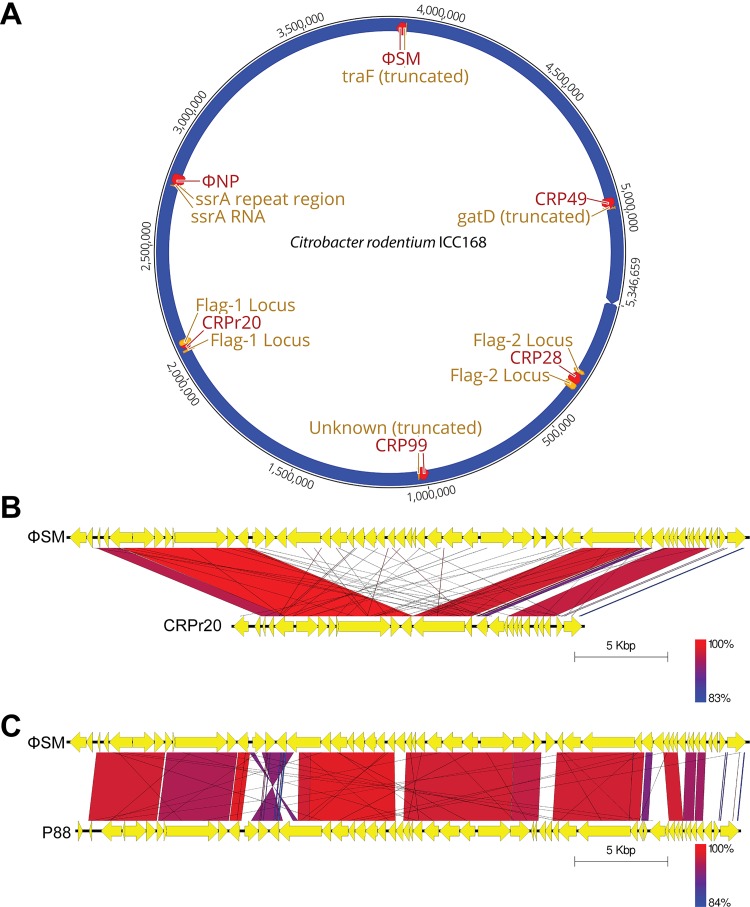
Prophage insertion sites in C. rodentium ICC168. (A) Genomic organization of the C. rodentium ICC168 prophages (red) and corresponding insertion sites (yellow). Note the integration of CRPr20 and CRP28 into the Flag-1 and -2 flagellar biosynthesis gene clusters, respectively, of the nonmotile C. rodentium genome. Remnant prophages CRPr11, CRPr13, CRPr17, and CRPr30 are not depicted due to insertion in noncoding regions. (B) Whole-genome alignment by blastn of CRPr20 and ΦSM. The high degree of sequence similarity suggests the potential for recombination between the active ΦSM and reservoir CRPr20. (C) Whole-genome alignment by blastn of P88 and ΦSM. Both represent active temperate phages isolated in enteric pathogens E. coli K88 and C. rodentium, respectively. DNA is represented by black bars with coding regions shown as arrows. Similarity was determined by blastn, with high (red) and low (blue) degrees of sequence match indicated by a color scale. Bar, 5 kbp.

While the function of the gene disrupted by CRP99 insertion remained unclear, our bioinformatic reassessment of the C. rodentium chromosome suggested a potential function for the previously hypothetical coding domain perturbed by ΦSM prophage integration (Fig. 6A). Unlike the case in E. coli K-12 but utilizing an identical *attB* sequence, in C. rodentium ΦSM integrates into the middle of a gene encoding a 421-amino-acid (aa) putative conjugal transfer protein with a conserved *traF* domain (pfam13729, demonstrating 95% identity to the conjugal transfer proteins of other species of *Citrobacter* and 85% amino acid identity to that of Salmonella enterica). TraF is a protein involved in the F-plasmid-specific type IV secretion system (T4SS) and is required for pilus assembly (44). The immediate upstream and downstream genomic regions surrounding *traF* as far as 100 kbp in each direction contain various metabolic and regulatory coding regions with no apparent coding regions associated with mobile genetic elements, F-specific conjugative factors, or integrative conjugative elements. It is unclear if this integration interferes with the capacity for F-specific plasmid conjugal transfer in C. rodentium as T4SS conjugation families can utilize elements from other conjugative pathways (45). However, any potential perturbation of mechanisms related to genetic transfer might help explain the conservation of 10 prophage regions, at least 2 of which, ΦNP and ΦSM, could functionally facilitate HGT.

Genomic alignment of the C. rodentium prophage regions to each other revealed ΦSM and CRPr20 to contain a high degree of sequence similarity, with 80% of CRPr20 sharing 99% nucleotide sequence identity with the 5′ and 3′ ends of ΦSM (with the other 20% of CRPr20 representing novel open reading frames predominantly found in the third quarter of the CRPr20 genome) (Fig. 6B). This suggested that CRPr20 might be the result of a partial ΦSM translocation or integration event back into the genome of C. rodentium. Notably, the deletion around the center of CRPr20 was found to occur at the juncture of ORF12 (corresponding to ORF12 in ΦSM), a predicted DNA invertase, and ORF13 (corresponding to ORF37 in ΦSM), a predicted recombinase (Data Set S1). The high level of relatedness between the two prophage regions suggests that genetic recombination could occur within the host genome. In this way, the defective CRPr20 might act as a genetic reservoir of ΦSM-facilitated horizontal acquisitions, providing an alternate storage repository for foreign coding domains within the C. rodentium chromosome. Such a mechanism has been described with the temperate phage λ, with relaxed homologous recombination occurring with defective prophage regions in E. coli that confer new accessory genes from/to each region (46). Similarly, in C. rodentium, this might allow for the maintenance of an important vector of horizontal genetic exchange while simultaneously facilitating the continued chromosomal incorporation of new genetic material.

### Reannotation of the C. rodentium prophage regions reveals cargo genes likely associated with evolution toward pathogenicity.

Our reannotation of the 10 C. rodentium prophages revealed the predicted function of 66 previously uncharacterized, hypothetical coding domains (Data Set S1, highlighted in green). While half of the annotated coding regions corresponded to protein products implicated in phage replication, assembly, or lysis, 32 genes encoded products of predicted function directly implicated in either known A/E virulence pathways or potential fitness-conferring capacities (Table 2). We identified a number of fitness factors, including such putative metabolic and housekeeping proteins as an oxidoreductase, a sulfate transporter, a serine protease, a member of the multidrug/metabolite transporter (DMT) superfamily, a sialate *O*-acetyltransferase, a nucleoside triphosphate pyrophosphohydrolase, a chromosome/plasmid partitioning protein, and a DNA damage-inducible protein as well as a number of non-phage-associated transcriptional regulators. While not directly linked to known virulence pathways in an obvious way, such functional duplications of native chromosomally encoded proteins might increase C. rodentium fitness within the mouse gut environment. In this way, these potential fitness factors might encourage prophage maintenance and conservation within the C. rodentium chromosome by counterbalancing seemingly nonbeneficial prophage protein products such as lytic enzymes.

**TABLE 2:** Prophage cargo genes associated with pathogenic evolution

Prophage and gene	Predicted protein function^*a*^	Predicted property^*b*^
ΦNP		
ORF1	DNA damage-inducible DinI-like protein	Fitness
ORF4	Serine acetyltransferase	Fitness
ORF24	Serine peptidase	Fitness
ORF28	DNA-directed RNA polymerase subunit σ^70^	Fitness
ORF43	GntR family transcriptional regulator	Fitness
ORF48	Putative lipoprotein	Virulence
ORF56	ATPase	Fitness
ORF59	ATPase	Fitness
ΦSM (formerly CRP38)		
ORF1	T6SS VasI family protein	Virulence
ORF31	Oxidoreductase	Fitness
ORF35	Plasmid stability and chromosome partitioning protein	Fitness
ORF42	MarR family transcriptional regulator	Fitness
CRP28		
ORF10	Multidrug DMT transporter permease	Fitness
ORF18	T6SS ATPase ClpV1 family protein	Virulence
ORF48	Sulfate transporter	Fitness
CRP49		
ORF41	Multidrug DMT transporter permease	Fitness
ORF51	Sialate *O*-acetyltransferase	Fitness
CRP99		
ORF28	Peptidase	Fitness
ORF37	Membrane-bound lytic murein transglycosylase	Fitness/virulence
ORF51	Nucleoside triphosphate pyrophosphohydrolase	Fitness
CRPr11		
ORF11	Primosomal protein N	Fitness
CRPr13		
ORF3	T3SS effector protein EspX7 (HECT-type E3 ubiquitin transferase)	Virulence
ORF4	T3SS secreted effector EspN-like protein	Virulence
ORF5	T3SS effector	Virulence
ORF6	T3SS effector E3 ubiquitin transferase SspH2-like protein	Virulence
CRPr17		
ORF1	T3SS secreted effector NleC-like protein	Virulence
CRPr20		
ORF16	Serine protease	Fitness
ORF19	MarR family transcriptional regulator	Fitness
CRPr33		
ORF3	T3SS effector NleG-like protein	Virulence
ORF4	T3SS effector NLeG8-like protein	Virulence
ORF5	T3SS effector NLeG8-like protein	Virulence
ORF6	IpaB/EvcA family *bfpT* chaperone-regulated protein	Virulence

a Determined by blastp and analysis of conserved domains.

b Coding products not classically ascribed to prophage regulation, assembly, structure, or lysis were categorized as either fitness or virulence (if the predicted gene product was known to play a role in pathogenicity) factors.

Bioinformatic interrogation on the predicted protein coding level also revealed several proteins directly linked with known virulence pathways. This included VasI family-type and ClpV family-type ATPases of type VI secretion systems (T6SS). The T6SS is a targeting machinery intended for the delivery of toxins into other bacteria and, as reported in Pseudomonas aeruginosa, eukaryotic host cells (47, 48). The T6SS of C. rodentium has been shown to confer a fitness advantage within microbial communities, and, indeed, throughout infection a decrease in the abundance of host-beneficial, probiotic *Lactobacillus* species can be seen in addition to other commensal die-off (49, 50). So, it is possible that the C. rodentium T6SS and these associated protein products are implicated in this step of pathogenesis, if not also in host infection.

In addition, we identified nine prophage-associated coding domains as T3SS effector proteins, most closely resembling the EHEC/EPEC-coded Esp- and Nle-type effectors. While multifunctional, acting in the subversion of a variety of host signaling and metabolic pathways, almost all Esp- and Nle-type effectors studied have proven to be essential virulence determinants (51). EPEC A/E lesion formation is dependent on non-LEE-encoded effectors (52). Importantly, ∼90% of coding regions within the C. rodentium prophage regions are actively transcribed under standard growth conditions, an observation marked as unusual for the normally tightly regulated expression associated with prophage elements (12). As such, it seems likely that the cargo genes predicted to be associated with virulence and fitness discussed above represent a collection of active pathogenicity determinants exogenous to the C. rodentium LEE and so may be promising candidates for future molecular and mechanistic study. Furthermore, a recent study showed that an EHEC O157:H7 strain 86-24 Stx-containing inducible prophage possessed a Cro-like transcription factor which activates both the EHEC and C. rodentium T3SS, enhancing virulence (53). This could suggest that seemingly innocuous non-phage-associated transcription factors and phage-regulatory coding genes might play a significant role in C. rodentium virulence as well.

### C. rodentium prophages are conserved across known human enteric pathogens in a horizontally acquired manner.

Last, we examined the prevalence of C. rodentium prophage regions within other bacterial species. If these prophage regions are directly implicated in C. rodentium’s evolution toward an enteric pathogenic lifestyle, they would likely represent a conserved group of genetic elements found in other bacterial pathogens and could act as possible genetic reservoirs for the evolution of future pathogens.

Bioinformatic analysis by nucleotide alignment revealed the conservation of C. rodentium prophage regions across a wide range of Enterobacteriaceae genomes (Table 3). With the exception of the ΦNP locus, conserved prophage regions showed relatively high rates of DNA sequence identity (∼80% on average). Notably, these conserved prophages maintained high degrees of similarity to the C. rodentium prophage regions encoding possible virulence and fitness factors, with primary differentiations observed around modules regulating phage structure and assembly. In almost all cases, we identified the strains containing similar prophage regions as pathogenic. These included strains of A/E virulent EPEC and EHEC as well as pathogenic strains of Salmonella enterica, Shigella boydii, Shigella sonnei, and Klebsiella pneumoniae. Only two of the identified strains were originally isolated from the mouse gut, including E. coli strains M8 and M10, with the majority having been isolated from human clinical isolates and most commonly from patients exhibiting diarrheagenic infection. This observation was consistent with the theory that C. rodentium evolved concurrently with the development of mouse models for human diseases (12) and suggested active cross-transfer between bacteria of human and mouse hosts. Deeper analysis of the previously alluded to EHEC O157:H7 strain 86-24 genome revealed a prophage (genome position, bp 2015661 to 2085889) that shares 40.1% identity to ΦSM. Although this region is not the Cro- and Stx-containing induced prophage region shown to enhance EHEC and C. rodentium virulence (53), this suggests another notable evolutionary link between C. rodentium and an A/E strain reliant upon genes carried by prophages for T3SS activity and virulence.

**TABLE 3:** Conservation of C. rodentium prophages

Prophage (%GC)^*a*^	Bacterial strain containing similar prophage^*b*^	GenBank accession no.	% identity	Bacterial/prophage %GC^*c*^	Source (pathogenic?)^*d*^
ΦNP (53.1)	Enterobacter cloacae complex Hoffmann cluster IV strain DSM 16690	CP017184.1	43.7	56.2/52.1	Human clinical isolate (yes)
	Salmonella enterica subsp. *enterica* serovar Saintpaul strain FDAARGOS_373	CP023512.1	25.7	52.2/49.1	Human clinical isolate (yes)
	Enterobacter lignolyticus strain G5	CP012871.1	25.2	57.3/53.0	Soil isolate (no)
	Enterobacter hormaechei strain CAV1176	CP011662.1	21.2	55.4/53.2	Human clinical isolate (yes)
ΦSM (52.2)	Escherichia coli strain IHE3034	CP001969.1	95.1	50.7/51.7	Human clinical isolate (yes)
	Escherichia coli strain UTI89	CP000243.1	81.5	50.6/52.6	Human clinical isolate (yes)
	Escherichia coli O104:H21 strain CFSAN002236	CP023541.1	80.6	50.7/51.1	Human clinical isolate (yes)
	Salmonella enterica subsp. *enterica* serovar Paratyphi A strain ATCC 1151	CP019185.1	79.4	52.2/52.6	Human clinical isolate (yes)
CRPr20 (52.5)	Escherichia coli strain MDR_56	CP019903.1	74.5	50.8/51.6	Human clinical isolate (yes)
	Escherichia coli strain M10	CP010200.1	70.1	50.9/52.2	Mouse isolate (no)
	Shigella boydii strain ATCC 9210	CP011511.1	44.6	51.2/53.5	Human clinical isolate (yes)
	Escherichia coli O145:H28 strain RM12581	CP007136.1	41.2	50.7/53.7	Agricultural isolate (yes)
CRP28 (53.9)	Escherichia coli O127:H6 EPEC1	LT903847.1	80.4	50.7/53.6	Human clinical isolate (yes)
	Escherichia coli strain M8	CP019953.1	76.3	50.6/53.7	*ob*/*ob* mouse isolate (yes)
	Escherichia coli strain Ecol_AZ161	CP018991.1	75.3	50.6/53.4	Human clinical isolate (yes)
	Escherichia coli O136:H16 strain 08-00022	CP013662.1	75.2	50.8/52.6	Agricultural isolate (yes)
CPR49 (53.6)	Escherichia coli strain CI5	CP011018.1	87.3	50.8/54.2	Human clinical isolate (yes)
	Shigella sonnei strain 2015C-3566	CP022457.1	84.5	52.0/54.4	Human clinical isolate (yes)
	Escherichia coli O157:H7 strain Sakai	NC_002695.1	78.5	50.5/53.4	Human clinical isolate (yes)
	Salmonella enterica subsp. *salamae* serovar 57:z29:z42	CP022467.1	56.3	52/54.9	Avian isolate (yes)
CRP99 (56.3)	Pantoea stewartii subsp. *stewartii* DC283	CP017581.1	81.9	54.2/56.6	Agricultural isolate (yes)
	Klebsiella pneumoniae strain AR_0107	CP021955.1	78.3	57.2/55.6	Human clinical isolate (yes)
	Klebsiella quasivariicola strain KPN1705	CP022823.1	77.4	56.1/58.1	Human clinical isolate (yes)
	Citrobacter farmeri strain AUSMDU00008141	CP022695.1	75.7	53.4/55.3	Human clinical isolate (yes)

a This table is a nonexhaustive list of the top four conserved prophage genomes corresponding to each C. rodentium prophage region; prophages CRPr11, CRPr13, CRPr17, and CRPr33 were not included in analysis due to heavy deletion of core prophage coding regions. %GC, percent GC content.

b Determined by blastn (https://blast.ncbi.nlm.nih.gov/Blast.cgi).

c Prophage regions of corresponding bacteria were determined by PHAST.

d Information according to NCBI BioSample identification associated with each genomic accession number.

We also compared the C. rodentium prophage regions to a database of viral genome sequences. Only ΦSM yielded a substantial match, showing 76.8% sequence identity to enterobacterial phage P88 (Fig. 6C). Phage P88 is a recently isolated P2-like phage generated by chemical induction of a porcine enterotoxigenic E. coli K88 prophage region and has been shown to be capable of lysing a wide range of E. coli strains (54). The K88 genome contains 40 genomic islands, totaling 814 kb (15%) in total genome content, and 15 of these islands are prophage-associated loci, including P88 (55). Studies have found portions of these islands in E. coli O157:H7 to be involved in colonization of ligated pig intestine (56). In this way, ΦSM is representative of a conserved, fully functional temperate phage found across at least two known enteric pathogens. It is likely that conserved ΦSM-like prophages are either spontaneously active, as with ΦSM, or could be induced, as with P88.

**FIG 7:**
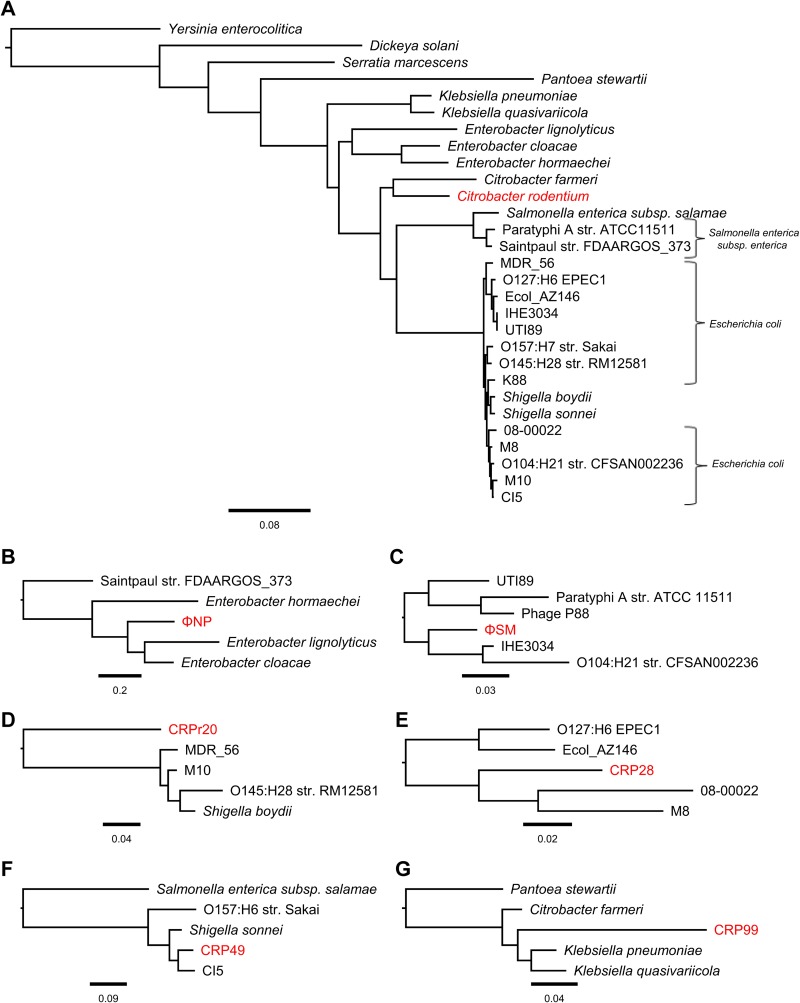
Phylogenetic analysis of the C. rodentium prophages. (A) Maximum likelihood tree of C. rodentium showing the phylogenetic relationship of C. rodentium to enteric bacteria containing conserved prophage regions (Table 3) based on the nucleotide sequences of seven housekeeping genes (*adk*, *fumC*, *gyrB*, *icd*, *mdh*, *purA*, and *recA*). The tree was rooted using the outgroups of *Yersinia, Serratia*, and *Dickeya*. (B to G) Maximum likelihood trees of the six predominantly intact C. rodentium prophages, as indicated, showing the phylogenetic relationship of the conserved prophage regions of the bacteria examined in panel A. No apparent consistency of backbone and prophage tree organizations was noted. Trees were constructed using PhyML with default parameters and the general time reversible (GTR) model with gamma distribution. Branch support was tested using an approximate likelihood ratio test (aLRT) based on the Shimodaira-Hasegawa-like (SH-like) procedures and retested using 1,000 bootstrap replicates. Tree structures and root positions were verified by Bayesian phylogenetic analysis using BEAST2 under a GTR substitution model, which yielded results consistent with PhyML. Trees are drawn to scale. Scale bars represent the number of substitutions per site. C. rodentium and its corresponding prophages are in red.

These data suggest that the C. rodentium prophages represent a set of genetic elements conserved across enteric bacteria that evolved toward pathogenicity. We next examined whether these prophage regions existed as a function of a shared pathogenic enteric backbone or represented horizontally acquired, mobile determinants of enteric pathogen evolution. We conducted a phylogenetic analysis utilizing the whole-genome sequences of C. rodentium and the strains listed in Table 3. First, we generated a multilocus sequence analysis (MLSA) based on the DNA sequences of seven conserved backbone genes (*adk, fumC, gyrB, icd, mdh, purA,* and *recA*) located in core regions of the genome and a maximum likelihood phylogenetic tree constructed with PhyML (57) and verified by Bayesian modeling with BEAST2 (58) (Fig. 7A). Our results were consistent with previous constructions of the Enterobacteriaceae phylogeny (42, 59). Next, the matching genomic prophage regions of the strains were determined using PHAST (60) and aligned to generate maximum likelihood trees corresponding to each C. rodentium prophage region (Fig. 7B to G). The branch organization of prophage phylogenetic trees diverges significantly from those of the backbones of corresponding prophage host genomes generated by MLSA, with no MLSA-consistent order of C. rodentium prophage lineage emerging from analysis. This observation was consistent with the typically mobile nature of prophage elements and the observed differing GC contents between corresponding prophages and hosts (Table 3). Moreover, it suggested that the prophages of C. rodentium are not associated with a common pathogenic enteric backbone, but, rather, they represent a family of horizontally acquired, enteric-bacterium-associated, and transferable pathogenicity determinants.

## DISCUSSION

C. rodentium is a natural host-adapted intestinal mouse pathogen and an important model organism for studying EPEC, EHEC, and A/E pathogenicity as well as a variety of intestinal disorders and diseases (10, 11). Previous studies focused predominantly on the examination of LEE-associated molecular mechanisms of host-bacterium infection and interaction (1). Little was known about the nature and importance of C. rodentium prophage-mediated virulence factors or their role in genetic exchange and intermicrobial effects in relation to infection and the murine gut microbiome. In this study, we examine all 10 identified prophage regions of C. rodentium and their potential roles in directing C. rodentium toward a pathogenic lifestyle.

An earlier study identified a fully functional active temperate prophage, phage ΦNP, which was spontaneously released by C. rodentium into the extracellular environment (12). Through characterization of ΦNP, we discovered a second novel and fully functional temperate phage encoded by CRP38, named ΦSM. The host ranges of both ΦNP and ΦSM were E. coli strain dependent with host recognition mediated by LPS in a GlcII and GlcIII residue-specific manner, respectively. It was also found that ΦSM was capable of infecting mutants possessing a GlcI residue lacking a Gal side chain, suggesting that this LPS structure, when lacking the Gal side chain and remaining outer core sugars, possesses similar chemistry to the GlcIII residue as “seen” by the phage. Interestingly, this observation might also suggest an evolved resistance to spontaneous host mutation of the LPS biosynthesis operon which might perturb the immediate and adjacent coding regions of *waaR* (product catalyzes addition of GlcIII), including the directly upstream *waaO* (product catalyzes addition of GlcII) and *waaB* (product catalyzes addition of Gal side chain) coding regions. The residue-specific manner of ΦNP and ΦSM LPS adsorption might also suggest that the C. rodentium phages evolved to allow for dual infection, as consistent with their heteroimmunity. Identification of common extracellular surface elements (LPS residues) as phage receptors, the nondeleterious integration of both ΦNP and ΦSM observed in K-12 lysogens, and the wide conservation of ΦNP and ΦSM *attB* loci suggested that ΦNP and ΦSM are important mediators of HGT in C. rodentium.

These observations may be pertinent to a recent model suggesting that C. rodentium relies on commensal gut microbiota for successful colonization of colonic mucosa (22). Expression of colonization and virulence factors of A/E pathogens is regulated by a variety of external stimuli, including microbiota-derived metabolites (61, 62). It is possible that C. rodentium prophage integration, viral gene expression, and phage-induced lysis, although nondeleterious in terms of the bacterial population, alter the metabolic profile of commensals, exacerbating enteric infection. Correspondingly, genetic exchange mediated by ΦNP and ΦSM might facilitate the acquisition of commensally encoded colonization determinants. As such, the role of CRPr20 as a likely reservoir of ΦSM-facilitated horizontal acquisitions might be implicated in a wider context than simply as a means of overcoming prophage-induced functional gene loss. It is currently unknown if ΦNP and/or ΦSM play active roles in gene transfer or metabolic interaction between enteric commensals and C. rodentium during colonization. Nevertheless, the characterization presented here of two fully functional prophages of C. rodentium with widely conserved integration sites that can replicate efficiently in E. coli strains provides an obvious and potentially facile route to the development of novel viral vectors for genome engineering and synthetic biology applications in this murine pathogen. This provides opportunities to enhance the value of experimental investigations using this tractable infection model for human enteric pathology.

Prophages can also play a direct role in bacterial pathogenicity by encoding virulence and fitness factors promoting access to a niche environment (18, 21). Our study revealed several C. rodentium prophage-encoded putative metabolic and housekeeping proteins not obviously associated with phage propagation or life cycle. Furthermore, several T6SS assembly pathway factors were identified as well as several T3SS EHEC/EPEC-homologue effector proteins (Esp and Nle type). Given that a previous study found that ∼90% of coding regions within the C. rodentium prophage regions were actively transcribed under standard growth conditions (12), these prophage-encoded factors are candidate pathogenicity determinants exogenous to the C. rodentium LEE and could be promising for future molecular and mechanistic study. With a recent analysis demonstrating the dependence of EPEC A/E lesion formation on non-LEE-encoded effectors (52), it is possible that these C. rodentium-encoded prophage effectors represent significant virulence determinants. It seems plausible that acquisition and maintenance of these prophages in C. rodentium were initial steps toward its host-adapted pathogenic lifestyle as these effectors and fitness factors would have helped facilitate access to a previously unfavorable niche, the murine gut. In the context of prophage insertion site location, the bacterium’s nonmotility due to prophage integration into key flagellar genomic sites might have furthered this evolutionary advantage by inhibiting a metabolically costly process (it is estimated that 2.1% of biosynthetic energy is utilized for flagellar biosynthesis processes in E. coli) (63). Moreover, the inherent motility provided by physiological peristalsis of the murine intestine and spread through fecal excretion could likely compensate for the loss of movement generated by flagellar biosynthesis disruption. In a similar fashion, prophage conservation and maintenance might have further been supported by the insertion of prophage elements, like ΦSM, into sites associated with forms of natively encoded HGT mechanisms, possibly increasing reliance on prophage-facilitated HGT for genetic diversification and adaptation in new environments.

Importantly, we observed conservation of these prophages across a wide range of Enterobacteriaceae genomes, with almost all strains examined identified as pathogenic, primarily to humans. We identified only three nonpathogenic bacterial genomes containing conserved prophage regions, two of which (E. coli M8 and M10) were murine isolates. This suggests that the C. rodentium prophages were predominantly representative of a family of genetic elements conserved across enteric bacteria that evolved toward pathogenicity. Phylogenetic analysis revealed that these conserved genetic regions were unlikely to be part of a common pathogenic enteric backbone but, rather, may represent a family of horizontally acquired enteric-bacterium-associated pathogenic determinants. While the acquisition of ΦNP- and ΦSM-like conserved prophages can be explained by their functional nature, it is less clear by which means the other prophage regions have propagated. However, as previously noted, the nonfunctional but intact prophages CRP28, CRP38, CRP48, and CRP99 had been shown to be induced and excised under standard LB growth conditions; this study also noted the presence variable genomic regions intermingled with these prophage elements, suggesting illegitimate excision if not active transposition (12). This could be a means by which horizontal transfer of these virulence determinant-associated prophages might have been facilitated. Alternatively, imprecise integration events could have rendered once-active phages incapable of forming functional virions. An earlier prokaryotic-wide phylogenetic study concluded that horizontal transfer, not duplication, drives the expansion of protein families and functionality in prokaryotes (59). As evidenced here, this model can be expanded to the evolution of enteric pathogens. Accordingly, similar systematic examinations of prophage elements in other pathogenic bacteria, including strains associated with outbreaks of human disease, might provide deeper evolutionary and pathological insights.

## MATERIALS AND METHODS

### Bacterial strains and culture conditions.

The bacterial strains used in this study are listed in Table 4. Strains of *Citrobacter*, Escherichia coli, *Salmonella,* and *Pseudomonas* were grown at 37°C; all other strains listed were grown at 30°C. Overnight cultures were grown in 5 ml of Luria broth (LB) in sterile 25-ml culture tubes placed on a rotary wheel. Bacterial growth was determined by measuring the optical density of the culture at a wavelength of 600 nm (OD_600_) using a Unicam Heλios spectrophotometer and cuvettes with a 1-cm path length. Solid medium contained 1.5% (wt/vol) agar with soft-medium overlay (top agar) using 0.35% agar; both were made with LB unless otherwise noted. For long-term storage, 800 μl of overnight cultures was mixed with 200 μl of 80% (wt/vol) vacuum-sterilized glycerol. Samples were briefly vortexed, appropriately labeled, and stored at −80°C. Phage buffer was composed of 10 mM Tris-HCl, pH 7.4, 10 mM MgSO_4_, and 0.01% gelatin. Phages used in this study are listed in Table 5.

**TABLE 4:** Bacterial strains used in this study

Strain	Relevant characteristics	Source or reference
Citrobacter rodentium ICC168	Reference strain	12
Escherichia coli		
MG1655	K-12 derivative; F^−^ λ^−^ *rph-1*	73
W3110	K-12 derivative; F^−^ λ^−^ IN(*rrnD-rrnE*)*1 rph-1*	74
ER2507	K-12 derivative; F^−^ *ara-14 leuB6 fhuA2 Δ*(*argF-lac*)*U169 lacY1 glnV44 galK2 rpsL20 xyl-5 mtl-5 Δ*(*malB*) *zjc*::Tn*5*(*kan*) *Δ*(*mcrC-mrr*)*HB101*	New England Biotech
DH5α	λ^−^ ϕ80d*lacZ*ΔM15 Δ(*lacZYA-argF*)*U169 recA1 endA hsdR17*(r_K_^−^ m_K_^−^) *supE44 thi-1 gyrA relA1*	Invitrogen
LE392	K-12 derivative; F*^−^ e14* mutant (*mcrA* mutant) *hsdR514*(r_K_^−^ m_K_^+^) *glnV44 supF58 lacY1 Δ*(*lacIZY*)*6 galK2 galT22 metB1 trpR55*	75
β2163 (pDS1028)	K-12 derivative, donor strain for random transposon mutagenesis containing pDS1028; F^−^ RP4-2-Tc::Mu Δ*dapA*::(*erm-pir*)	67
cc118 λpir	Laboratory-maintained K-12 derivative used for replicon cloning; *phoA20 thi-1 rspE rpoB argE*(*Am*) *recA1* λpir^+^	76
MG1655(ΦNP)	MG1655 ΦNP lysogen	This study
MG1655(ΦSM)	MG1655 ΦSM lysogen	This study
ER2507(ΦNP)	ER2507 ΦNP lysogen	This study
ER2507(ΦSM)	ER2507 ΦSM lysogen	This study
ER2507(ΦNP+ΦSM)	ER2507 dual ΦNP and ΦSM	This study
BW25113	K-12 derivative; *rrnB3 ΔlacZ4787 hsdR514 Δ*(*araBAD*)*567 Δ*(*rhaBAD*)*568 rph‐1*	41
JW3601-3	BW25113 Δ*waaR*::*kan*	41
JW3602-1	BW25113 Δ*waaO*::*kan*	41
JW3606-1	BW25113 Δ*waaG*::*kan*	41
JW3603-2	BW25113 *ΔwaaB*::*kan*	41
TY0703	BW25113 Δ*waaO‐waaB*::*cm*	38
TY0707	BW25113 Δ*waaF*::*cm*	38
TY0708	BW25113 Δ*waaC*::*cm*	38
TY0721	BW25113 Δ*waaR* Δ*ompC*::*kan*	38
TY0722	BW25113 Δ*waaO* Δ*ompC*::*kan*	38
TY0723	BW25113 Δ*waaO‐waaB*::*cm* Δ*ompC*::*kan*	38
TY0726	BW25113 Δ*waaG* Δ*ompC*::*kan*	38
TY0727	BW25113 Δ*waaF*::*cm* Δ*ompC*::*kan*	38
TY0728	BW25113 Δ*waaC*::*cm* Δ*ompC*::*kan*	38
JW0146-2	BW25113 *ΔfhuA*::*kan*	41
JW0401-1	BW25113 *Δtsx*::*kan*	41
JW0912-1	BW25113 *ΔompF*::*kan*	41
JW0940-6	BW25113 *ΔompA*::*kan*	41
JW1248-2	BW25113 *ΔompW*::*kan*	41
JW1607-1	BW25113 *ΔuidC*::*kan*	41
JW2203-1	BW25113 Δ*ompC*::*kan*	41
JW2341-1	BW25113 *ΔfadL*::*kan*	41
JW3619-1	BW25113 *ΔyicC*::*kan*	41
JW3996-1	BW25113 *ΔlamB*::*kan*	41
Serratia marcescens		
DB10	Wild type; nonpigmented	77
Sma395	Wild type	Clinical isolate
Mu378	Wild type	Clinical isolate
Msu497	Wild type	Clinical isolate
S421	Wild type	Clinical isolate
Others		
Citrobacter freundii Ballerup 7851	Wild type; Vi capsule positive	M. Popoff, Institut Pasteur
Dickeya solani MK10	Wild type	78
Kluyvera cryocrescens 2Kr27	Wild type	79
Pantoea agglomerans 10Bp14	Wild type	80
*Pectobacterium astrosepticum* SCRI-1043	Wild type	81
Photorhabdus luminescens subsp. *laumondii* TT01	Wild type	82
Pseudomonas aeruginosa PA01	Wild type	83
Salmonella enterica serovar Typhimurium	Wild type	Sanger Institute strain collection
*Serratia* sp. strain ATCC 39006	Wild type	84

**TABLE 5:** Bacteriophages used in this study

Phage	Description	Origin	Reference
ΦNP	Temperate phage of C. rodentium	C. rodentium supernatant	12
ΦSM	Temperate phage of C. rodentium	C. rodentium supernatant	This study
T4	Virulent coliphage	Lab stock	24
				λ*vir*	Virulent mutant of the temperate coliphage λ	Lab stock	23

### Phage and lysogen isolation.

ΦNP and ΦSM were isolated by titration of chloroform-treated supernatants of C. rodentium overnight cultures on appropriate K-12 host strain top lawns grown at 37°C. Single plaques were then picked with a sterile toothpick into 1.5-ml Eppendorf tubes containing 120 μl of phage buffer and 20 μl of chloroform, vortexed, spun down, and then titrated on LB agar (LBA) top lawns at 37°C. Lysogen strains were isolated by spotting 10 μl of high-titer phage lysates on LBA plates containing top agar overlays of the relevant bacterial host at 37°C. A sterile toothpick was then used to pick cells from turbid spots, and cells were then struck out onto LBA plates and allowed to grow overnight at 37°C. Lysogen identity was then confirmed by resistance to viral superinfection and spontaneous release of phages into the supernatant.

### Phage host range determination and effectiveness of plating.

Top agar overlays on LBA plates of the bacterial strain to be tested were spotted with a 10-μl sample of a high-titer lysate of the relevant phage, allowed to dry, and grown overnight at the temperature optimal for the bacterial strain. As a dilution control, 10-μl spots of only phage buffer were also added. Bacterial strains showing turbid clearance were then titrated in serial dilution in top agar overlays to achieve single, isolated plaques to confirm permissive host range. Efficiency of plating (EOP) was calculated as a ratio of the apparent titer of phage on the control strain to the observed phage titer on the test strain.

### Phage genomic DNA extraction.

Phage DNA was extracted using a phenol-chloroform protocol. In a phase-lock gel (PLG) tube, 450 μl of high-titer phage lysate was incubated with 4.5 μl of 1 mg/ml DNase I and 2.5 μl of 10 mg/ml RNase A and incubated at 37°C for 30 min. The mixture was then added to 11.5 μl of 20% SDS and 4.5 μl of 10 mg/ml proteinase K and incubated for another 30 min. DNA was extracted by adding 500 μl of a phenol-chloroform-isoamyl alcohol 25:24:1 mix and centrifuged at 1,500 × *g* for 5 min. The supernatant was transferred to a new PLG tube, and the previous step was repeated. In a new PLG tube, the supernatant was supplemented with 500 μl of chloroform-isoamyl alcohol at 24:1 and centrifuged at 1,500 × *g* for 5 min. The aqueous phase at the top was then incubated with 45 μl of sodium acetate (3 mol/liter, pH 5.2) and 500 μl of 100% isopropanol at room temperature for 45 min. The mixture was then subjected to centrifugation at 12,000 × *g* for 20 min, after which the pellet was washed at least twice with 70% ethanol and then resuspended in distilled H_2_O (dH_2_O).

### Transmission electron microscopy.

Glow-discharged, carbon-coated copper grids (obtained from the Multi-imaging Center, Department of Anatomy, University of Cambridge) were placed on 5-μl drops of high-titer phage to be imaged for 5 min. They were then washed briefly with water droplets three times before being blotted dry with sterile filter paper and placed on 5 μl of either 2% (wt/vol) phosphotungstic acid (PTA) for 10 min or uranyl acetate (UA) for 1 min. Grids were blotted to remove excess liquid, allowed to air dry, and visualized in a Phillips Tecnai G2 80- to 200-kV transmission electron microscope.

### Phage chemical mutagenesis.

Chemical mutagenesis was conducted utilizing hydroxylamine containing phosphate-EDTA buffer as previously described (64).

### Transfection of phage gDNA.

Transfection of viral gDNA into chemically competent cells was performed as previously described (65). As a control, λ*vir* gDNA was used. Transfected cells were examined for virus production by spinning down the transfectant recovery culture and removing 100 μl of the supernatant, treating it with chloroform, and titrating the crude supernatant lysate on top lawns of the relevant host species. This was repeated every hour for 3 h. Single plaques were counted and the number of PFU/(milliliter ·microgram of DNA) was determined.

### Structural phage proteome analysis.

Following purification by PEG 8000 and CsCl gradient, phage particles were analyzed by SDS-PAGE as previously described (24). Mass spectrometry was carried out at the University of Cambridge, Department of Biochemistry, according to the protocol noted on the departmental website (http://www3.bioc.cam.ac.uk/pnac/proteomics.html).

### Lysogen growth and phage release.

Overnight bacterial cultures to be tested were diluted to an OD_600_ of 0.05 in 25 ml of prewarmed LB or M9 minimal medium in 250-ml conical flasks and incubated at 37°C in a water bath with shaking at 185 rpm. Samples (1 ml) were taken every 30 min, and bacterial growth (optical density) was measured. Concurrently, 100-μl samples were collected into 1.5-ml Eppendorf tubes containing 900 μl of phage buffer and 30 μl of chloroform, vortexed, and stored at 4°C. Each time point sample was then titrated on two bacterial top lawns, containing either E. coli DH5α or ER2507 (as ΦNP can form plaques only onto ER2507, DH5α served to differentiate viral titer in the dual lysogen and served as a control for single-lysogen species). Plates with single plaques were then assessed, and the number of PFU/milliliter was recorded as a function of time.

### DNA manipulations, oligonucleotides, and sequencing.

Unless otherwise stated, standard molecular biological methods were used for all DNA manipulations. Genomic and plasmid DNA were purified using a GeneJET Genomic DNA purification kit (Thermo Scientific) and GeneJET Plasmid Miniprep kit (Thermo Scientific) according to manufacturer’s instructions. All restriction enzymes used were obtained from New England Biolabs and used according to the manufacturer’s protocols. DNA fragments were ligated using T4 DNA ligase (NEB). Oligonucleotides were obtained from Sigma-Aldrich and are listed in Table 6. DNA sequencing of PCR and plasmid products was performed by GATC Biotech utilizing their LightRun Tube barcodes for Sanger equencing.

**TABLE 6:** Oligonucleotides used in this study

Name	Sequence (5′–3′)	Comment(s)	Reference or source
SM.P67	TATCGAACTGCACCCGCAG	Forward primer; prophage CRP38 bp 15000	This study
SM.P69	GGTTCTGGCAACCATACTCATG	Reverse primer; prophage CRP38 bp 17250	This study
SM.P70a	AGAGGCTGGCTTGATTG	Primer for round 1 of RP-PCR for ΦNP	This study
SM.P70b	TACTGGTGCTGTCCTTG	Nested primer for round 2 of RP-PCR for ΦNP	This study
SM.P71a	TCAGATTAAACAGCAGTTTG	Primer for round 1 of RP-PCR for ΦSM	This study
SM.P71b	TGTCAGAGACTGAAAAAGG	Nested primer for round 2 of RP-PCR for ΦSM	This study
PF106	GACCACACGTCGACTAGTGCNNNNNNNNNNAGAG	RP-PCR primer 1	66
PF107	GACCACACGTCGACTAGTGCNNNNNNNNNNACGCC	RP-PCR primer 2	66
PF108	GACCACACGTCGACTAGTGCNNNNNNNNNNGATAC	RP-PCR primer 3	66
PF109	GACCACACGTCGACTAGTGC	RP-PCR adapter primer	66
oREM7	CTAGAGTCGACCTGCAGGC	pDS1028 replicon clone sequencing primer	67
SM.P22	GCCATAGCATTTTTATCC	Forward primer; pBAD33 sequencing primer	This study
SM.P23	TGATTTAATCTGTATCAGGC	Reverse primer; pBAD33 sequencing primer	This study
SM.P96	ACTGGTGGTGAGCTCGGTACAAAGAGGAGAAAACTAGATGCAGCAGGTGTTTT	Forward primer; *waaO* with RBS plus SacI cut site	This study
SM.P97	TACCTGCAGGTCGACGGATCCTTTAATGCTTTATCTTTTCAATAAA	Reverse primer; *waaO* with SalI cut site	This study
SM.P98	ACTGGTGGTGAGCTCGGTACAAAGAGGAGAAAACTAGGTGGACTCATTTCCTGC	Forward primer; *waaR* with RBS plus SacI cut site	This study
SM.P99	TACCTGCAGGTCGACGGATCCTTTATTTACGGTAATATTTTCGG	Reverse primer; *war* with SalI cut site	This study

### Random-primed PCR.

The random-primed PCR (RP-PCR) protocol was based on that previously described (66). In brief, colony or standard PCR was performed using the random primers PF106, PF107, and PF108 together with a phage-specific primer (Table 6). The reaction mix and PCR program used for the first round are described below. A second round of PCR amplification using the product of the first round as the template was performed under the same RP-PCR conditions using the primers PF109 and the nested phage-specific primer.

### Generation of plasmids used in this study.

The plasmids used in this study are listed in Table 7. LPS genes for complementation assays were amplified from E. coli K-12 gDNA using primers (Table 6) designed with 19 to 22 bp of complement and containing flanking regions holding either the SacI cut site and ribosomal binding site (RBS) or the SalI cut site. pBAD33 and these fragments were then digested with SacI and SalI, ligated, and used to transform the relevant K-12 strain. Successful transformants were then selected using appropriate antibiotic selection, sequence verified using primers SM.P22 and SM.P23, and moved into appropriate mutant background.

**TABLE 7:** Plasmids used in this study

Plasmid	Relevant characteristic(s)	Reference
pDS1028	Cm^r^ Tc^r^ Tnp oriR6K vector used for random transposon mutagenesis	67
pBAD33	Arabinose-inducible Cm^r^ plasmid	85
pBAD33(*waaO*)	pBAD33 E. coli K-12 *waaO*^+^	This study
pBAD33(*waaR*)	pBAD33 E. coli K-12 *waaR*^+^	This study

### Generation and screening of random transposon mutants.

Using E. coli β2165 (pDS1028) as a donor strain and ER2507 as a recipient strain, generation of a random transposon insertion mutant library was carried out as previously described (67). In brief, a 30-μl conjugation mix of 1:3 donor/recipient cells was spotted onto a plate containing LBA plus 2,6,-diaminopimelic acid (DAP), allowed to dry, and grown overnight. The mating patch was then harvested in 1 ml of LB, washed twice to remove residual DAP, and plated on LBA-Cm plates. Single colonies were then picked into 96-well plates containing 200 μl of LB-Cm and screened for phage resistance.

Mutant ER2507 cells were first screened for phage resistance in a mini-top-lawn format. Using a Corning 12-well tissue culture flat-bottom plate, 2 ml of LBA-Cm was added to each well. After this, mini-top lawns containing 10 μl of the phage to be tested (of the appropriate titer to see single plaques), 20 μl of the mutant to be tested, and 400 μl of top agar were overlaid onto each well containing LBA-Cm, and cultures were grown overnight. Mutant strains demonstrating resistance to plaque formation were then noted and further assessed with full-size plate assays in serial dilution. Those demonstrating full resistance or partial resistance were subjected to replicon cloning.

### Replicon cloning of transposon mutants.

Replicon cloning of pDS1028 ER2057 transposon insertion mutants was carried out as previously described (67). In brief, gDNA extractions of mutants to be sequenced were digested with restriction enzymes that were unable to cut within the transposed region (typically, XmnI and StuI were used [NEB]), ligated, and cloned into E. coli cc118 λ*pir*-containing cells. Transformants were then streaked out for purification and grown in liquid culture with the appropriate antibiotic; the plasmid was then extracted and sequenced using primer oREM7 (Table 6).

### Adsorption test.

Triplicate overnight cultures of strains to be tested were grown up in 5 ml of LB on a tube roller at 37°C. Premade Eppendorf tubes containing 900 μl of phage buffer and 30 μl of chloroform were labeled and set out for each sample and time point to be collected. To each sample of 5 ml of overnight bacterial culture of a high-titer phage was added to obtain a multiplicity of infection (MOI) of 0.01 and mixed immediately. From these mixtures 100 μl was removed for time point 0 min and added to the premade sample tubes and quickly vortexed for 5 s. Sample collection occurred in a similar fashion for the next 55 min every 5 to 10 min. Vortexed samples were then spun down, and supernatant was removed. These supernatants were then titrated in serial dilution on bacterial top lawns. The final adsorption curve was plotted by calculating the percentage of free phages in the culture against time. An LB-only sample was infected with phages as a negative control. In instances of complementation, all samples were induced with 0.1% arabinose at mid-log phase and allowed to grow for 3 h or until they reached an OD_600_ of 1.0 before use in adsorption studies.

### Bioinformatic analysis.

Coding sequences and ORFs were determined by a combination of prior annotations and the Geneious R7 predictive ORF function on known C. rodentium IIC168 genome and prophage sequences (GenBank accession number NC_013716) (68). Protein functionality and homology were predicted using BLASTP (https://blast.ncbi.nlm.nih.gov/Blast.cgi). Genome comparisons were generated by the Artemis Comparison Tool (ACT) (69) and EasyFig (70). Prophage regions were determined using PHAST (60), and PhiSITE (33) was used to determine bacteriophage regulatory units.

### Phylogenetic analysis.

Multiple-locus sequence analysis was conducted by individually extracting seven core housekeeping genes (*adk, fumC, gyrB, icd, mdh, purA,* and *recA*) from bacterial genomes of interest, including *Yersinia*, *Dickeya*, and *Serratia* outgroups. These genes were then individually aligned using ClustalW (71), and the alignments were concatenated and cured using Gblock (72). Phylogenetic trees were constructed by the maximum likelihood method using the general time reversible (GTR) model with gamma distribution in PhyML (57) and verified using Bayesian likelihood using the GTR model plus gamma with BEAST2 (58). Branch support was tested using an approximate likelihood ratio test (aLRT) based on the Shimodaira-Hasegawa-like (SH-like) procedures and retested using 1,000 bootstrap replicates. Assessment of conserved prophage regions was conducted using a similar workflow. Root positions of both MLSA and prophage were verified using Bayesian modeling.

## Supplementary Material

Supplemental file 1

Supplemental file 2

## References

[1] Collins JW, Keeney KM, Crepin VF, Rathinam VA, Fitzgerald KA, Finlay BB, Frankel G 2014 *Citrobacter rodentium:* infection, inflammation and the microbiota. Nat Rev Microbiol 12:612–623. doi:10.1038/nrmicro3315. 25088150

[2] Schauer DB, Falkow S 1993 Attaching and effacing locus of a *Citrobacter freundii* biotype that causes transmissible murine colonic hyperplasia. Infect Immun 61:2486–2492. 8500884 10.1128/iai.61.6.2486-2492.1993 PMC280873

[3] Barthold SW, Coleman GL, Jacoby RO, Livestone EM, Jonas AM 1978 Transmissible murine colonic hyperplasia. Vet Pathol 15:223–236. doi:10.1177/030098587801500209. 664189

[4] Wong ARC, Pearson JS, Bright MD, Munera D, Robinson KS, Lee SF, Frankel G, Hartland EL 2011 Enteropathogenic and enterohaemorrhagic *Escherichia coli*: even more subversive elements. Mol Microbiol 80:1420–1438. doi:10.1111/j.1365-2958.2011.07661.x. 21488979

[5] Mundy R, Petrovska L, Smollett K, Simpson N, Wilson RK, Yu J, Tu X, Rosenshine I, Clare S, Dougan G, Frankel G 2004 Identification of a novel *Citrobacter rodentium* type III secreted protein, EspI, and roles of this and other secreted proteins in infection. Infect Immun 72:2288–2302. doi:10.1128/IAI.72.4.2288-2302.2004. 15039354 PMC375195

[6] Nataro JP, Kaper JB 1998 Diarrheagenic *Escherichia coli*. Clin Microbiol Rev 11:142–201. doi:10.1128/CMR.11.1.142. 9457432 PMC121379

[7] Riley LW, Remis RS, Helgerson SD, McGee HB, Wells JG, Davis BR, Hebert RJ, Olcott ES, Johnson LM, Hargrett NT, Blake PA, Cohen ML 1983 Hemorrhagic colitis associated with a rare *Escherichia coli* serotype. N Engl J Med 308:681–685. doi:10.1056/NEJM198303243081203. 6338386

[8] Deng W, de Hoog CL, Yu HB, Li Y, Croxen MA, Thomas NA, Puente JL, Foster LJ, Finlay BB 2010 A comprehensive proteomic analysis of the type III secretome of *Citrobacter rodentium*. J Biol Chem 285:6790–6800. doi:10.1074/jbc.M109.086603. 20034934 PMC2825473

[9] Deng W, Li Y, Vallance BA, Finlay BB 2001 Locus of enterocyte effacement from *Citrobacter rodentium*: Sequence analysis and evidence for horizontal transfer among attaching and effacing pathogens. Infect Immun 69:6232–6235. doi:10.1128/IAI.69.10.6323-6335.2001. PMC98768 11553577

[10] Mundy R, MacDonald TT, Dougan G, Frankel G, Wiles S 2005 *Citrobacter rodentium* of mice and man. Cell Microbiol 7:1697–1706. doi:10.1111/j.1462-5822.2005.00625.x. 16309456

[11] Crepin VF, Collins JW, Habibzay M, Frankel G 2016 *Citrobacter rodentium* mouse model of bacterial infection. Nat Protoc 11:1851–1876. doi:10.1038/nprot.2016.100. 27606775

[12] Petty NK, Feltwell T, Pickard D, Clare S, Toribio AL, Fookes M, Roberts K, Monson R, Nair S, Kingsley RA, Bulgin R, Wiles S, Goulding D, Keane T, Corton C, Lennard N, Harris D, Willey D, Rance R, Yu L, Choudhary JS, Churcher C, Quail MA, Parkhill J, Frankel G, Dougan G, Salmond GPC, Thomson NR 2011 *Citrobacter rodentium* is an unstable pathogen showing evidence of significant genomic flux. PLoS Pathog 7:e1002018. doi:10.1371/journal.ppat.1002018. 21490962 PMC3072379

[13] Wommack KE, Colwell RR 2000 Virioplankton: viruses in aquatic ecosystems. Microbiol Mol Biol Rev 64:69–114. doi:10.1128/MMBR.64.1.69-114.2000. 10704475 PMC98987

[14] Echols H 1972 Developmental pathways for the temperate phage: lysis vs lysogeny. Annu Rev Genet 6:157–190. doi:10.1146/annurev.ge.06.120172.001105. 4604314

[15] Salmond GPC, Fineran PC 2015 A century of the phage: past, present and future. Nat Rev Microbiol 13:777–786. doi:10.1038/nrmicro3564. 26548913

[16] Mokrousov I 2009 *Corynebacterium diphtheriae*: genome diversity, population structure and genotyping perspectives. Infect Genet Evol 9:1–15. doi:10.1016/j.meegid.2008.09.011. 19007916

[17] Raffestin S, Marvaud JC, Cerrato R, Dupuy B, Popoff MR 2004 Organization and regulation of the neurotoxin genes in *Clostridium botulinum* and *Clostridium tetani*. Anaerobe 10:93–100. doi:10.1016/j.anaerobe.2004.01.001. 16701505

[18] Waldor M, Mekalanos J 1996 Lysogenic conversion by a filamentous phage encoding cholera toxin. Science 272:1910–1914. doi:10.1126/science.272.5270.1910. 8658163

[19] Tyler JS, Beeri K, Reynolds JL, Alteri CJ, Skinner KG, Friedman JH, Eaton KA, Friedman DI 2013 Prophage induction is enhanced and required for renal disease and lethality in an EHEC mouse model. PLoS Pathog 9:e1003236. doi:10.1371/journal.ppat.1003236. 23555250 PMC3610611

[20] Fortier L-C, Sekulovic O 2013 Importance of prophages to evolution and virulence of bacterial pathogens. Virulence 4:354–365. doi:10.4161/viru.24498. 23611873 PMC3714127

[21] Brussow H, Canchaya C, Hardt WD 2004 Phages and the evolution of bacterial pathogens: from genomic rearrangements to lysogenic conversion. Microbiol Mol Biol Rev 68:560–602. doi:10.1128/MMBR.68.3.560-602.2004. 15353570 PMC515249

[22] Mullineaux-Sanders C, Collins JW, Ruano-Gallego D, Levy M, Pevsner-Fischer M, Glegola-Madejska IT, Sågfors AM, Wong JLC, Elinav E, Crepin VF, Frankel G 2017 *Citrobacter rodentium* relies on commensals for colonization of the colonic mucosa. Cell Rep 21:3381–3389. doi:10.1016/j.celrep.2017.11.086. 29262319 PMC5746604

[23] Hendrix RW, Duda RL 1992 Bacteriophage lambda PaPa: not the mother of all lambda phages. Science 258:1145–1148. doi:10.1126/science.1439823. 1439823

[24] Laemmli UK 1970 Cleavage of structural proteins during the assembly of the head of bacteriophage T4. Nature 227:680–685. doi:10.1038/227680a0. 5432063

[25] Lima-Mendez G, Toussaint A, Leplae R 2011 A modular view of the bacteriophage genomic space: identification of host and lifestyle marker modules. Res Microbiol 162:737–746. doi:10.1016/j.resmic.2011.06.006. 21767638

[26] Nanda AM, Thormann K, Frunzke J 2015 Impact of spontaneous prophage induction on the fitness of bacterial populations and host-microbe interactions. J Bacteriol 197:410–419. doi:10.1128/JB.02230-14. 25404701 PMC4285972

[27] De Paepe M, Tournier L, Moncaut E, Son O, Langella P, Petit MA 2016 Carriage of λ latent virus is costly for its bacterial host due to frequent reactivation in monoxenic mouse intestine. PLoS Genet 12:e1005861. doi:10.1371/journal.pgen.1005861. 26871586 PMC4752277

[28] Little JW, Michalowski CB 2010 Stability and instability in the lysogenic state of phage lambda. J Bacteriol 192:6064–6076. doi:10.1128/JB.00726-10. 20870769 PMC2976446

[29] Yamamoto Y, Sunohara T, Jojima K, Inada T, Aiba H 2003 SsrA-mediated trans-translation plays a role in mRNA quality control by facilitating degradation of truncated mRNAs. RNA 9:408–418. doi:10.1261/rna.2174803. 12649493 PMC1370408

[30] Pease AJ, Roa BR, Luo W, Winkler ME 2002 Positive growth rate-dependent regulation of the *pdxA*, *ksgA*, and *pdxB* genes of *Escherichia coli* K-12. J Bacteriol 184:1359–1369. doi:10.1128/JB.184.5.1359-1369.2002. 11844765 PMC134838

[31] Hockney RC, Scott TA 1979 The isolation and characterization of three types of vitamin B6 auxotrophs of *Escherichia coli* K12. J Gen Microbiol 110:275–283. doi:10.1099/00221287-110-2-275. 374678

[32] Shine J, Dalgarno L 1974 The 3′-terminal sequence of *Escherichia coli* 16S ribosomal RNA: complementarity to nonsense triplets and ribosome binding sites. Proc Natl Acad Sci U S A 71:1342–1346. doi:10.1073/pnas.71.4.1342. 4598299 PMC388224

[33] Klucar L, Stano M, Hajduk M 2010 PhiSITE: database of gene regulation in bacteriophages. Nucleic Acids Res 38:D366–D370. doi:10.1093/nar/gkp911. 19900969 PMC2808901

[34] Browning DDF, Busby SJWS 2004 The regulation of bacterial transcription initiation. Nat Rev Microbiol 2:57–65. doi:10.1038/nrmicro787. 15035009

[35] Silva JB, Storms Z, Sauvageau D 2016 Host receptors for bacteriophage adsorption. FEMS Microbiol Lett 363:fnw002. doi:10.1093/femsle/fnw002. 26755501

[36] Tzipilevich E, Habusha M, Ben-Yehuda S 2017 Acquisition of phage sensitivity by bacteria through exchange of phage receptors. Cell 168:186–199. e12. doi:10.1016/j.cell.2016.12.003. 28041851

[37] Henning U, Jann K 1979 Two-component nature of bacteriophage T4 receptor activity in *Escherichia coli* K-12. J Bacteriol 137:664–666. 368036 10.1128/jb.137.1.664-666.1979 PMC218498

[38] Washizaki A, Yonesaki T, Otsuka Y 2016 Characterization of the interactions between *Escherichia coli receptors*, LPS and OmpC, and bacteriophage T4 long tail fibers. Microbiologyopen 5:1003–1015. doi:10.1002/mbo3.384. 27273222 PMC5221442

[39] Wang X, Quinn PJ 2010 Lipopolysaccharide: biosynthetic pathway and structure modification. Prog Lipid Res 49:97–107. doi:10.1016/j.plipres.2009.06.002. 19815028

[40] Stevenson G, Neal B, Liu D, Hobbs M, Packer NH, Batley M, Redmond JW, Lindquist L, Reeves P 1994 Structure of the O antigen of *Escherichia coli* K-12 and the sequence of its *rfb* gene cluster. J Bacteriol 176:4144–4156. doi:10.1128/jb.176.13.4144-4156.1994. 7517391 PMC205614

[41] Baba T, Ara T, Hasegawa M, Takai Y, Okumura Y, Baba M, Datsenko KA, Tomita M, Wanner BL, Mori H 2006 Construction of *Escherichia coli* K-12 in-frame, single-gene knockout mutants: the Keio collection. Mol Syst Biol 2:2006.0008. doi:10.1038/msb4100050. PMC1681482 16738554

[42] Petty NK, Bulgin R, Crepin VF, Cerdeño-Tárraga AM, Schroeder GN, Quail MA, Lennard N, Corton C, Barron A, Clark L, Toribio AL, Parkhill J, Dougan G, Frankel G, Thomson NR 2010 The *Citrobacter rodentium* genome sequence reveals convergent evolution with human pathogenic *Escherichia coli*. J Bacteriol 192:525–538. doi:10.1128/JB.01144-09. 19897651 PMC2805327

[43] Ren CP, Beatson SA, Parkhill J, Pallen MJ 2005 The Flag-2 locus, an ancestral gene cluster, is potentially associated with a novel flagellar system from *Escherichia coli*. J Bacteriol 187:1430–1440. doi:10.1128/JB.187.4.1430-1440.2005. 15687208 PMC545627

[44] Arutyunov D, Arenson B, Manchak J, Frost LS 2010 F plasmid TraF and TraH are components of an outer membrane complex involved in conjugation. J Bacteriol 192:1730–1734. doi:10.1128/JB.00726-09. 20081027 PMC2832511

[45] Guglielmini J, de la Cruz F, Rocha EPC 2013 Evolution of conjugation and type IV secretion systems. Mol Biol Evol 30:315–331. doi:10.1093/molbev/mss221. 22977114 PMC3548315

[46] De Paepe M, Hutinet G, Son O, Amarir-Bouhram J, Schbath S, Petit MA 2014 Temperate phages acquire DNA from defective prophages by relaxed homologous recombination: the role of Rad52-like recombinases. PLoS Genet 10:e1004181. doi:10.1371/journal.pgen.1004181. 24603854 PMC3945230

[47] Coulthurst SJ 2013 The type VI secretion system—a widespread and versatile cell targeting system. Res Microbiol 164:640–654. doi:10.1016/j.resmic.2013.03.017. 23542428

[48] Sana TG, Berni B, Bleves S 2016 The T6SSs of *Pseudomonas aeruginosa* strain PAO1 and their effectors: beyond bacterial-cell targeting. Front Cell Infect Microbiol 6:6:61. doi:10.3389/fcimb.2016.00061. 27376031 PMC4899435

[49] Hoffmann C, Hill DA, Minkah N, Kirn T, Troy A, Artis D, Bushman F 2009 Community-wide response of the gut microbiota to enteropathogenic *Citrobacter rodentium* infection revealed by deep sequencing. Infect Immun 77:4668–4678. doi:10.1128/IAI.00493-09. 19635824 PMC2747949

[50] Gueguen E, Cascales E 2013 Promoter swapping unveils the role of the *Citrobacter rodentium* CTS1 type VI secretion system in interbacterial competition. Appl Environ Microbiol 79:32–38. doi:10.1128/AEM.02504-12. 23064344 PMC3536073

[51] Dean P, Kenny B 2009 The effector repertoire of enteropathogenic *E. coli*: ganging up on the host cell. Curr Opin Microbiol 12:101–109. doi:10.1016/j.mib.2008.11.006. 19144561 PMC2697328

[52] Cepeda-Molero M, Berger CN, Walsham ADS, Ellis SJ, Wemyss-Holden S, Schüller S, Frankel G, Fernández LÁ 2017 Attaching and effacing (A/E) lesion formation by enteropathogenic *E. coli* on human intestinal mucosa is dependent on non-LEE effectors. PLoS Pathog 13:e1006706. doi:10.1371/journal.ppat.1006706. 29084270 PMC5685641

[53] Hernandez-Doria JD, Sperandio V 2018 Bacteriophage transcription factor Cro regulates virulence gene expression in enterohemorrhagic *Escherichia coli*. Cell Host Microbe 23:607–617.e6. doi:10.1016/j.chom.2018.04.007. 29746832 PMC5982111

[54] Chen M, Zhang L, Xin S, Yao H, Lu C, Zhang W 2017 Inducible prophage mutant of *Escherichia coli* can lyse new host and the key sites of receptor recognition identification. Front Microbiol 8:147. doi:10.3389/fmicb.2017.00147. 28203234 PMC5285337

[55] Shepard SM, Danzeisen JL, Isaacson RE, Seemann T, Achtman M, Johnson TJ 2012 Genome sequences and phylogenetic analysis of K88- and F18-positive porcine enterotoxigenic *Escherichia coli*. J Bacteriol 194:395–405. doi:10.1128/JB.06225-11. 22081385 PMC3256668

[56] Yin X, Wheatcroft R, Chambers JR, Liu B, Zhu J, Gyles CL 2009 Contributions of O island 48 to adherence of enterohemorrhagic *Escherichia coli* O157:H7 to epithelial cells in vitro and in ligated pig ileal loops. Appl Environ Microbiol 75:5779–5786. doi:10.1128/AEM.00507-09. 19633120 PMC2747874

[57] Guindon S, Gascuel O 2003 A simple, fast, and accurate algorithm to estimate large phylogenies by maximum likelihood. Syst Biol 52:696–704. doi:10.1080/10635150390235520. 14530136

[58] Bouckaert R, Heled J, Kühnert D, Vaughan T, Wu CH, Xie D, Suchard MA, Rambaut A, Drummond AJ 2014 BEAST 2: a software platform for Bayesian evolutionary analysis. PLoS Comput Biol 10:e1003537. doi:10.1371/journal.pcbi.1003537. 24722319 PMC3985171

[59] Treangen TJ, Rocha EPC 2011 Horizontal transfer, not duplication, drives the expansion of protein families in prokaryotes. PLoS Genet 7:e1001284. doi:10.1371/journal.pgen.1001284. 21298028 PMC3029252

[60] Zhou Y, Liang Y, Lynch KH, Dennis JJ, Wishart DS 2011 PHAST: a fast phage search tool. Nucleic Acids Res 39:W347–W352. doi:10.1093/nar/gkr485. 21672955 PMC3125810

[61] Curtis MM, Hu Z, Klimko C, Narayanan S, Deberardinis R, Sperandio V 2014 The gut commensal *Bacteroides thetaiotaomicron* exacerbates enteric infection through modification of the metabolic landscape. Cell Host Microbe 16:759–769. doi:10.1016/j.chom.2014.11.005. 25498343 PMC4269104

[62] De Nisco NJ, Rivera-Cancel G, Orth K 2018 The biochemistry of sensing: enteric pathogens regulate type III secretion in response to environmental and host cues. mBio 9:e02122-17. doi:10.1128/mBio.02122-17. 29339429 PMC5770552

[63] Zhao K, Liu M, Burgess RR 2007 Adaptation in bacterial flagellar and motility systems: from regulon members to “foraging”-like behavior in *E. coli*. Nucleic Acids Res 35:4441–4452. doi:10.1093/nar/gkm456. 17576668 PMC1935009

[64] Villafane R 2009 Construction of phage mutants. Methods Mol Biol 501:223–237. doi:10.1007/978-1-60327-164-6_20. 19066824

[65] Seto H, Tomasz A 1974 Early stages in DNA binding and uptake during genetic transformation of pneumococci. Proc Natl Acad Sci U S A 71:1493–1498. doi:10.1073/pnas.71.4.1493. 4151520 PMC388256

[66] Fineran PC, Everson L, Slater H, Salmond GPC 2005 A GntR family transcriptional regulator (PigT) controls gluconate-mediated repression and defines a new, independent pathway for regulation of the tripyrrole antibiotic, prodigiosin, in *Serratia*. Microbiology 151:3833–3845. doi:10.1099/mic.0.28251-0. 16339930

[67] Monson R, Smith DS, Matilla MA, Roberts K, Richardson E, Drew A, Williamson N, Ramsay J, Welch M, Salmond GPC 2015 A plasmid-transposon hybrid mutagenesis system effective in a broad range of enterobacteria. Front Microbiol 6:1442. doi:10.3389/fmicb.2015.01442. 26733980 PMC4686594

[68] Kearse M, Moir R, Wilson A, Stones-Havas S, Cheung M, Sturrock S, Buxton S, Cooper A, Markowitz S, Duran C, Thierer T, Ashton B, Meintjes P, Drummond A 2012 Geneious basic: an integrated and extendable desktop software platform for the organization and analysis of sequence data. Bioinformatics 28:1647–1649. doi:10.1093/bioinformatics/bts199. 22543367 PMC3371832

[69] Carver TJ, Rutherford KM, Berriman M, Rajandream MA, Barrell BG, Parkhill J 2005 ACT: The Artemis comparison tool. Bioinformatics 21:3422–3423. doi:10.1093/bioinformatics/bti553. 15976072

[70] Sullivan MJ, Petty NK, Beatson SA 2011 EasyFig.: a genome comparison visualizer. Bioinformatics 27:1009–1010. doi:10.1093/bioinformatics/btr039. 21278367 PMC3065679

[71] Thompson JD, Higgins DG, Gibson TJ 1994 CLUSTAL W: Improving the sensitivity of progressive multiple sequence alignment through sequence weighting, position-specific gap penalties and weight matrix choice. Nucleic Acids Res 22:4673–4680. doi:10.1093/nar/22.22.4673. 7984417 PMC308517

[72] Castresana J 2000 Selection of conserved blocks from multiple alignments for their use in phylogenetic analysis. Mol Biol Evol 17:540–552. doi:10.1093/oxfordjournals.molbev.a026334. 10742046

[73] Blattner FR, Plunkett G, Bloch CA, Perna NT, Burland V, Riley M, Collado-Vides J, Glasner JD, Rode CK, Mayhew GF, Gregor J, Davis NW, Kirkpatrick HA, Goeden MA, Rose DJ, Mau B, Shao Y 1997 The complete genome sequence of *Escherichia coli* K-12. Science 277:719–720. 10.1126/science.277.5331.1453 9278503

[74] Jensen KF 1993 The *Escherichia coli* K-12 ‘wild types’ W3110 and MG1655 have an *rph* frameshift mutation that leads to pyrimidine starvation due to low *pyrE* expression levels. J Bacteriol 175:3401–3407. doi:10.1128/jb.175.11.3401-3407.1993. 8501045 PMC204738

[75] Sambrook J, Frisch E, Maniatis T 1989 Molecular cloning: a laboratory manual. Cold Spring Harbor Laboratory Press, Cold Spring Harbor, NY.

[76] Herrero M, De Lorenzo V, Timmis KN 1990 Transposon vectors containing non-antibiotic resistance selection markers for cloning and stable chromosomal insertion of foreign genes in gram-negative bacteria. J Bacteriol 172:6557–6567. doi:10.1128/jb.172.11.6557-6567.1990. 2172216 PMC526845

[77] Flyg C, Kenne K, Boman HG 1980 Insect pathogenic properties of *Senatia marcescens*: phageresistant mutants with a decreased resistance to *Cecropia* immunity and a decreased virulence to *Drosophila. J*. Gen Microbiol 120:173–181. doi:10.1099/00221287-120-1-173. 7012273

[78] Garlant L, Koskinen P, Rouhiainen L, Laine P, Paulin L, Auvinen P, Holm L, Pirhonen M 2013 Genome sequence of *Dickeya solani*, a new soft rot pathogen of potato, suggests its emergence may be related to a novel combination of non-ribosomal peptide/polyketide synthetase clusters. Diversity 5:824–842. doi:10.3390/d5040824.

[79] Farmer JJ, Fanning GR, Huntley-Carter GP, Holmes B, Hickman FW, Richard C, Brenner DJ 1981 *Kluyvera*, a new (redefined) genus in the family Enterobacteriaceae: identification of *Kluyvera ascorbata* sp. nov. and *Kluyvera cryocrescens* sp. nov. in clinical specimens. J Clin Microbiol 13:919–933. 7240403 10.1128/jcm.13.5.919-933.1981 PMC273917

[80] Delétoile A, Decré D, Courant S, Passet V, Audo J, Grimont P, Arlet G, Brisse S 2009 Phylogeny and identification of *Pantoea* species and typing of *Pantoea agglomerans* strains by multilocus gene sequencing. J Clin Microbiol 47:300–310. doi:10.1128/JCM.01916-08. 19052179 PMC2643697

[81] Gardan L, Gouy C, Christen R, Samson R 2003 Elevation of three subspecies of *Pectobacterium carotovorum* to species level: *Pectobacterium atrosepticum* sp. nov., *Pectobacterium betavasculorum* sp. nov. and *Pectobacterium wasabiae* sp. nov. Int J Syst Evol Microbiol 53:381–391. doi:10.1099/ijs.0.02423-0. 12710602

[82] Duchaud E, Rusniok C, Frangeul L, Buchrieser C, Givaudan A, Taourit S, Bocs S, Boursaux-Eude C, Chandler M, Charles J-F, Dassa E, Derose R, Derzelle S, Freyssinet G, Gaudriault S, Médigue C, Lanois A, Powell K, Siguier P, Vincent R, Wingate V, Zouine M, Glaser P, Boemare N, Danchin A, Kunst F 2003 The genome sequence of the entomopathogenic bacterium *Photorhabdus luminescens*. Nat Biotechnol 21:1307–1313. doi:10.1038/nbt886. 14528314

[83] Stover CK, Pham XQ, Erwin AL, Mizoguchi SD, Warrener P, Hickey MJ, Brinkman FS, Hufnagle WO, Kowalik DJ, Lagrou M, Garber RL, Goltry L, Tolentino E, Westbrock-Wadman S, Yuan Y, Brody LL, Coulter SN, Folger KR, Kas A, Larbig K, Lim R, Smith K, Spencer D, Wong GK, Wu Z, Paulsen IT, Reizer J, Saier MH, Hancock RE, Lory S, Olson MV 2000 Complete genome sequence of *Pseudomonas aeruginosa* PA01, an opportunistic pathogen. Nature 406:959–964. doi:10.1038/35023079. 10984043

[84] Monson RE, Tashiro Y, Salmond GPC 2016 Overproduction of individual gas vesicle proteins perturbs flotation, antibiotic production and cell division in the enterobacterium *Serratia* sp. ATCC 39006. Microbiology 162:1595–1607. doi:10.1099/mic.0.000347. 27519819

[85] Guzman LM, Belin D, Carson MJ, Beckwith J 1995 Tight regulation, modulation, and high-level expression by vectors containing the arabinose P(BAD) promoter. J Bacteriol 177:4121–4130. doi:10.1128/jb.177.14.4121-4130.1995. 7608087 PMC177145

