# Supplementary material for: The Prophages of Citrobacter rodentium Represent a Conserved Family of Horizontally Acquired Mobile Genetic Elements Associated with Enteric Evolution towards Pathogenicity
Source: J Bacteriol. 2019 Apr 9;201(9):e00638-18. doi: 10.1128/JB.00638-18 (PMC6456863; doi:10.1128/JB.00638-18)
Supplement: Supplemental file 1 [file JB.00638-18-s0001.pdf]

Supporting Information

SData 1. Overview of reannotated *C. rodentium* prophages. Collection of the reannotated *C. rodentium* prophage open reading frames (ORFs). ORFs were collated, translated according to standard bacterial codons, and assessed using blastp across known or predicted proteins within the NCBI BLAST database. Predicted functions were assigned based upon protein domain (pfam) and/or amino acid sequence consensus to known or predicted proteins. The bacterial species containing the protein of highest consensus identity to each prophage ORF is listed as well as the accession number of that homologous match. In addition, the percent identity and correlating number of amino acid matches are listed. ORF numbers were assigned in an ascending 5' to 3' direction as according to the submitted NCBI GenBank sequence for *C. rodentium* ICC168 (Accession: NC_013716.1). ORFs with functions identified for first time through this studied are noted in green. ORFs of unknown/hypothetical function are noted in yellow.

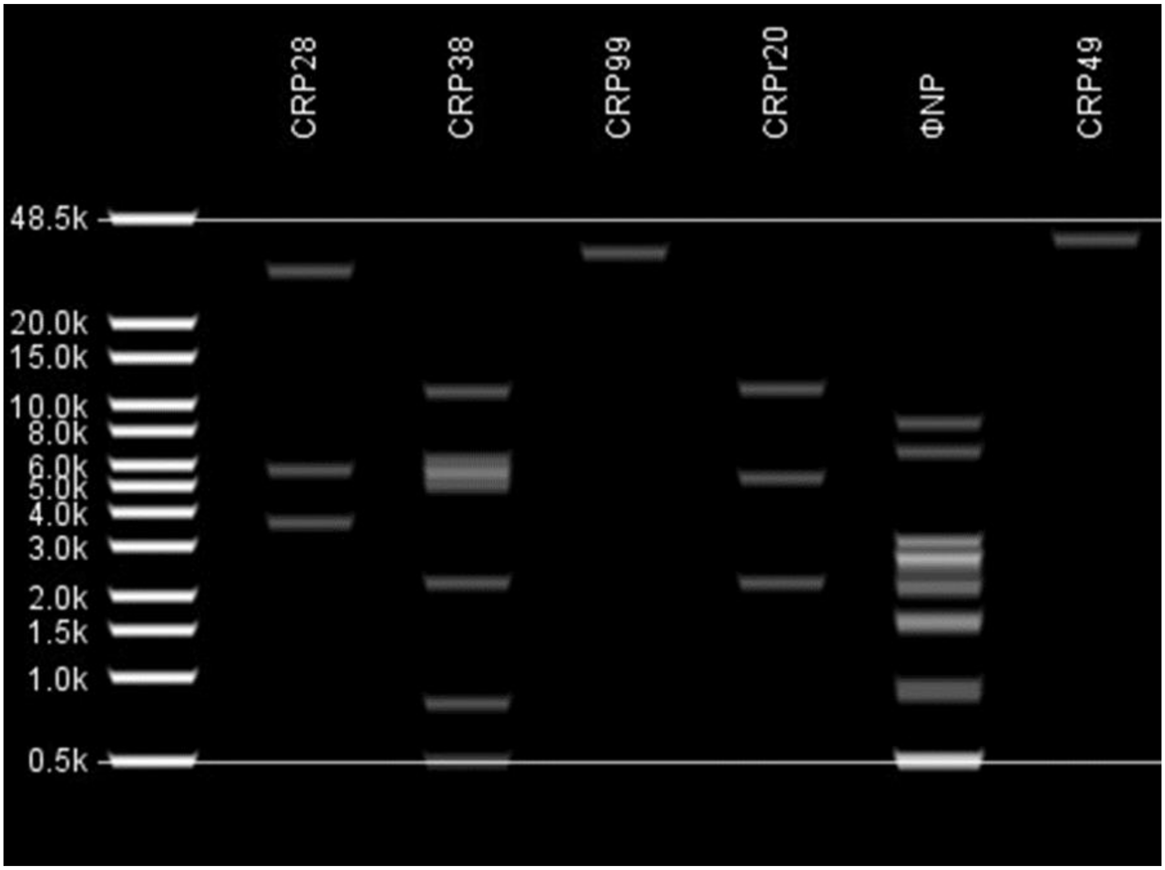

SFig. 1. Virtual gel of digested prophage genomes. Virtual gel showing the predicted cut patterns of BamHI for the intact *C. rodentium* prophages CRP28, CRP38, CRP49, CRP99, CRPr20, and ΦNP. Prophages CRPr11, CRPr13, CRPr17, and CRPr33 were not included in analysis due to heavy deletion of core prophage coding regions. Note how the cut pattern for DH5α and ER2507 found in Fig1D match those predicted for CRP38 and ΦNP, respectively, here. Both CRP99 and CRP49 lack any BamHI cutsites. This image was made using Geneious R11.

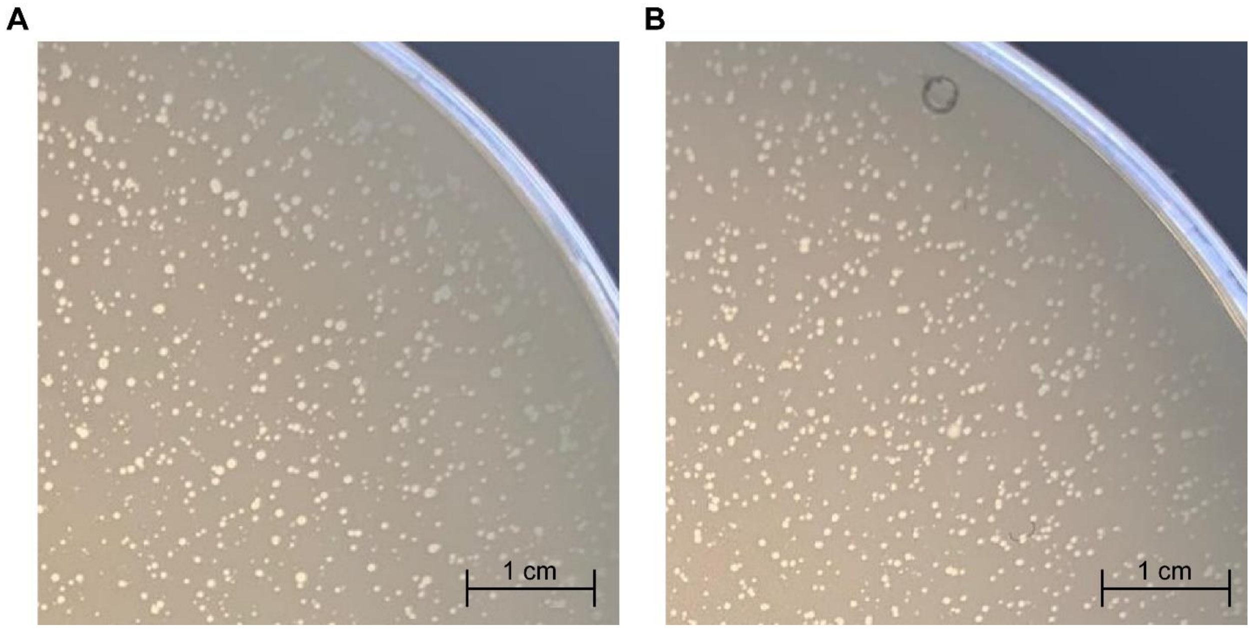

SFig. 2. The plaque morphologies of ΦNP and ΦSM. (A) ΦNP and (B) ΦSM generate identical pin-point, turbid plaques when assayed in an *E. coli* K-12 agar top lawn. Shown is an *E. coli* K-12 str. ER2507 agar top lawn containing plaques generated by ΦSM. Scale bar is 1 cm.

A

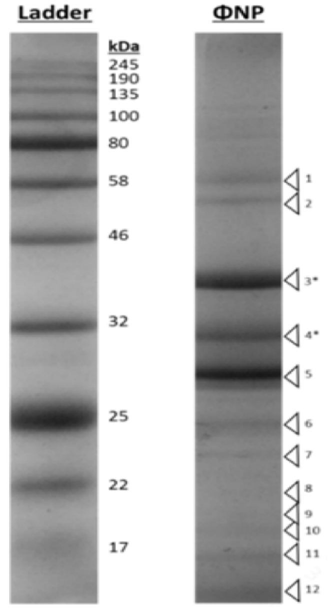

B

Band	Approximate Size (kDa)	Predicted ORF	Predicted Size (kDa)	Predicted Structure
**1**	62	25	61.47	Portal protein
**2**	54	17	53.03	Tail sheath protein
**3***	38	21*	38.94	Major capsid protein
**4***	31	50*	31.46	Unknown
**5**	29	6	29.38	Tail protein
**6**	25	30	25.01	Unknown
**7**	23	29	23.74	Unknown
**8**	22	7	21.85	Tail protein
**9**	19	23	21.36	Unknown
**10**	18	9	16.82	Unknown
**11**	15	22	14.41	Head decoration protein
**12**	14	49	13.39	Unknown

C

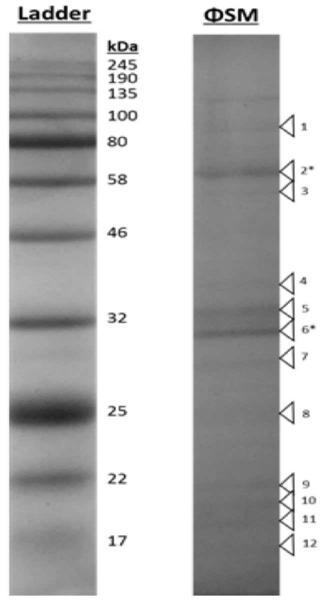

D

Band	Approximate Size (kDa)	Predicted ORF	Predicted Size (kDa)	Predicted Structure
**1**	100	10	99.8	Tail tape measure protein
**2***	65	16	64.86	Tail fiber protein
**3**	52	5	46.3	Tail protein
**4**	38	6	43.52	Tail sheath protein
**5**	35	32	39.28	Portal protein
**6***	32	29	39.2	Major capsid protein
**7**	29	30	30.64	Capsid scaffolding protein
**8**	25	13	25.83	Tail protein
**9**	20	17	19.89	Tail protein
**10**	19	7	18.8	Major tail tube protein
**11**	18	11	18.41	Tail protein
**12**	16	8	13.48	Tail protein

SFig. 3. Structural proteomes of ΦNP and ΦSM. (A, C) SDS-PAGE analysis of purified ΦNP (A) and ΦSM (C) particles adjacent to broad range ladder (ladder and phage were run on same gel and cropped to insert annotations). The gel was stained with Coomassie, photographed, and analyzed with ImageJ software. (C, D) Predicted size and function of annotated bands. ΦNP bands 3* and 4* and ΦSM bands 2* and 3* were verified by mass spectrometry with the remainder being predicted based upon migration and predicted size.

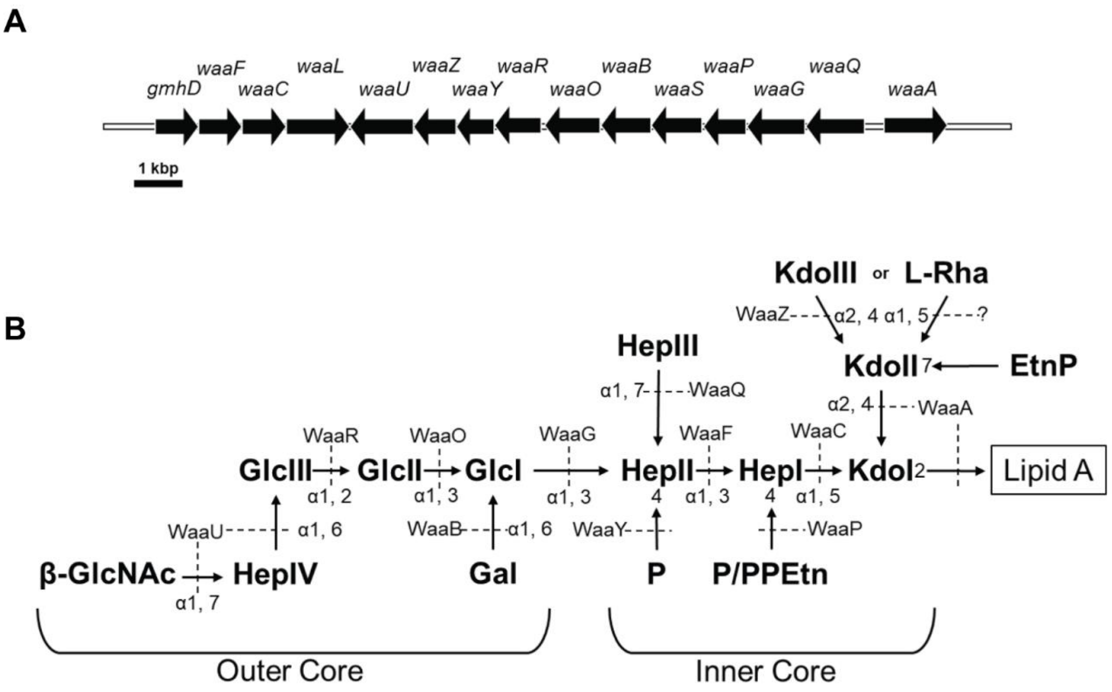

SFig. 4. The *E. coli* K-12 LPS biosynthetic pathway. (A) Genetic organization of the *E. coli* K-12 LPS biosynthetic cluster (scale bar 1 kbp). (B) Structure and assembly pathways of the K-12 LPS. Horizontal arrows indicate main chain residues with vertical arrows denoting sugar-branches. Dotted lines note the reactions catalyzed by the known or predicted sugar transferases with corresponding glycosidic linkages labeled. [Abbreviations: Glc, glucose; Gal, galactose; β-GlcNAc, β-N-acetylglucosamine; Kdo, 3-deoxy-D-manno-oct-2-ulosonic acid; Hep, heptose; Rha, rhamnose; EtnP, ethanolamine phosphate; PPEtn, 2-aminoethyl diphosphate; and P, phosphate.
